# Economic burden of antibiotic resistance in ESKAPE organisms: a systematic review

**DOI:** 10.1186/s13756-019-0590-7

**Published:** 2019-08-13

**Authors:** Xuemei Zhen, Cecilia Stålsby Lundborg, Xueshan Sun, Xiaoqian Hu, Hengjin Dong

**Affiliations:** 1 Center for Health Policy Studies, School of Public Health, Zhejiang University School of Medicine, 866 Yuhangtang Road, Hangzhou, 310058 Zhejiang China; 2 Global Health-Health Systems and Policy (HSP): Medicines, focusing antibiotics, Department of Public Health Sciences, Karolinska Institutet, Stockholm, Sweden; 3 The Fourth Affiliated Hospital Zhejiang University School of Medicine, No. N1, Shancheng Avenue, Yiwu City, Zhejiang China

**Keywords:** Antibiotic resistance, Economic burden, ESKAPE organism, *S. aureus*, *Enterococcus*, *A. baumannii*, *E. coli*, *K. pneumoniae*, *P. aeruginosa*

## Abstract

**Background:**

Antibiotic resistance (ABR) is one of the biggest threats to global health. Infections by ESKAPE (*Enterococcus*, *S. aureus*, *K. pneumoniae*, *A. baumannii*, *P. aeruginosa*, and *E. coli*) organisms are the leading cause of healthcare-acquired infections worldwide. ABR in ESKAPE organisms is usually associated with significant higher morbidity, mortality, as well as economic burden. Directing attention towards the ESKAPE organisms can help us to better combat the wide challenge of ABR, especially multi-drug resistance (MDR).

**Objective:**

This study aims to systematically review and evaluate the evidence of the economic consequences of ABR or MDR ESKAPE organisms compared with susceptible cases or control patients without infection/colonization in order to determine the impact of ABR on economic burden.

**Methods:**

Both English-language databases and Chinese-language databases up to 16 January, 2019 were searched to identify relevant studies assessing the economic burden of ABR. Studies reported hospital costs (charges) or antibiotic cost during the entire hospitalization and during the period before/after culture among patients with ABR or MDR ESKAPE organisms were included. The costs were converted into 2015 United States Dollars. Disagreements were resolved by a third reviewer.

**Results:**

Of 13,693 studies identified, 83 eligible studies were included in our review. The most studied organism was *S. aureus*, followed by *Enterococcus*, *A. baumannii*, *E. coli*, *E. coli* or/and *K. pneumoniae*, *P. aeruginosa*, and *K. pneumoniae*. There were 71 studies on total hospital cost or charge, 12 on antibiotic cost, 11 on hospital cost or charge after culture, 4 on ICU cost, 2 on hospital cost or charge before culture, and 2 on total direct and indirect cost. In general, ABR or MDR ESKAPE organisms are significantly associated with higher economic burden than those with susceptible organisms or those without infection or colonization. Nonetheless, there were no differences in a few studies between the two groups on total hospital cost or charge (16 studies), antibiotic cost (one study), hospital cost before culture (one study), hospital cost after culture (one study). Even, one reported that costs associated with MSSA infection were higher than the costs for similar MRSA cases.

**Conclusions:**

ABR in ESKAPE organisms is not always, but usually, associated with significantly higher economic burden. The results without significant differences may lack statistical power to detect a significant association. In addition, study design which controls for severity of illness and same empirical antibiotic therapy in the two groups would be expected to bias the study towards a similar, even negative result. The review also highlights key areas where further research is needed.

**Electronic supplementary material:**

The online version of this article (10.1186/s13756-019-0590-7) contains supplementary material, which is available to authorized users.

## Background

Antibiotics have been pivotal in treating and preventing common infections, but the laws of evolution and natural selection along with the overuse and misuse have contributed to an alarming increase in antibiotic resistance (ABR) worldwide. As the selection of antibiotics is getting smaller together with only slow changes in prescription behavior, we are heading for a post-antibiotic era [1]. ABR is one of the biggest threats to global health, endangering not only the achievements towards the Millennium Development Goals but also the Sustainable Development Goals [2]. ABR is usually associated with significant higher morbidity, mortality, prolongation of illness and reduced labour efficiency [3–9]. In high-income countries, it was estimated that ABR resulted in as much as $20 billion in excess direct costs, with $35 billion in societal costs for lost productivity each year in the United States (US) alone [10]. In the European Union (EU) and European Economic Area (EEA) countries, a subset of ABR organisms is associated with extra healthcare costs and lost productivity amounting to €1.1–1.5 billion yearly if there is no prompt and effective action [11, 12]. Globally, it would lose 1.1–3.8% of its annual gross domestic product (GDP) due to antimicrobial resistance (AMR) by 2050 [13]. Low- and middle-income countries will suffer more [14]. Countries in the sub-Saharan Africa may face a GDP loss of 0.1–2.5% [14]. A total of 24 million people would be forced into extreme poverty due to AMR by 2030, especially in low-income countries [13].

To combat ABR, the World Health Organization (WHO) in 2015 published a global action plan, it is expected that individual countries will develop their own national action plans on AMR in keeping with this global plan. However, the absence of economic assessments on economic burden of ABR is an obstacle to implementation of global or national strategies for containment of ABR [15]. It is necessary to conduct economic research to assess the costs of ABR and the costs and benefits of global, national or regional action plans [15].

The WHO also developed a global priority list of ABR organisms to guide the research, discovery, and development of new antibiotics [16]. In this list, *Enterococcus spp.*, *Staphylococcus aureus*, *Klebsiella pneumoniae*, *Acinetobacter baumannii*, *Pseudomonas aeruginosa*, and *Escherichia coli*, collectively termed ESKAPE, have been identified as being increasingly involved in infectious diseases in humans. There were reports of third-generation cephalosporins resistance and fluoroquinolone resistance in *E. coli* exceeding 50% in five out of six WHO regions (African region, region of the Americas, Eastern Mediterranean region, European region, South-East Asia region, and Western Pacific region). For *K. pneumonia*, the six WHO regions had more than 50% resistance to third-generation cephalosporins and two WHO regions had more than 50% resistance to carbapenems. The overall Methicillin resistant *S. aureus* (MRSA) proportions exceeded 20% in the six WHO regions, and even exceeded 80% in three WHO regions [17].

ESKAPE organisms are the leading cause of healthcare-associated infections all over the world [18], especially in critically ill and immunocompromised individuals. These organisms consistently “escape” the effects of commonly used antibiotics and are a critical threat to public health [19]. Focusing attention on these pathogenic organisms is important since some studies have shown that patients with ABR ESKAPE organisms are more likely to receive inappropriate antibiotic therapy resulting in higher mortality rates and opportunities for spreading to other patients [20–23].

Several studies have examined the economic outcomes of resistant ESKAPE organisms in general and multi-drug resistance (MDR) specifically, but there has not been an in-depth, comparative analysis of the contemporary literature reporting on costs associated with resistant versus susceptible cases. In this study, we aimed to analyze the published literature of the economic consequences of resistant or MDR ESKAPE organisms compared with susceptible cases or control patients without infection/colonization.

## Methods

### Literature search

We performed a systematic search in the English-language databases (PubMed, Web of Science, and Embase) and Chinese-language databases (China National Knowledge Infrastructure, Wanfang data, and Chongqing VIP) up to January 16, 2019. In addition, we also manually reviewed the references from retrieved studies to ensure inclusion of all published studies. Detailed search strategies are provided in Additional file 1.

### Study selection

Inclusion and exclusion criteria were predefined. Inclusion criteria included (1) studies published in English or Chinese language; (2) publication date between January 1, 2000 and January 16, 2019; (3) original research of any type (cohort, case control, or observational study); (4) reports on humans; (5) reports on ESKAPE organisms; (6) reports on resistant versus susceptible cases or those without infection or colonization; and (7) reports on economic burden. Studies published before 2000 were not considered to ensure that the analysis focuses on contemporary literature that reflects current resistance patterns and clinical practice guidelines [4, 5]. Studies reporting on a group organisms (e.g. Gram-positive organisms, Gram-negative organisms, *Enterobacteriaceae*, *Enterobacter species*, etc.) were excluded as well. Both *E. coli* and *K. pneumonia* are members of *Enterobacteriaceae*, sharing characteristics and were therefor analyzed together. Two reviewers independently evaluated studies for eligibility based on titles and abstracts, then, reviewed the full text to decide if it met the inclusion criteria. Disagreements were resolved by a third reviewer.

### Data extraction

We developed a standardized extraction form to record the characteristics of each study, including first author, publication year, type of study, method, country, study setting, study period, study population, type of infection, type of hospital ward, organisms, sample size (cases and controls). Regarding the costs, population-adjusted costs were showed because that susceptible rates are more frequent than resistant rate, thus, susceptible organisms will cause more infections than resistant ones, in general. We extracted the currency and cost year, total hospital costs (charges) or antibiotic cost in median or mean values, and the statistical analysis of the cost differences. Costs were converted into 2015 US dollars using average exchange rates, then inflating this to 2015 currency estimates using the annual consumer price index [24, 25]. Hospital charge was defined as the amount that patient is expected to pay for care. Hospital cost was defined as expenses incurred by a hospital in providing patient care, including the sum of hospital charges and the amount from the reimbursement service. Again, disagreements were resolved by the third reviewer.

### Study quality assessment

The Newcastle-Ottawa quality assessment Scale (NOS) for cohort and case-control studies was used to assess study quality. A “star system” was developed to judge the study on three broad perspectives: (1) selection population (four items); (2) comparability of the groups (one item); and (3) ascertainment of either the exposure or outcome of interest (three items). The highest study quality in NOS was nine “stars” where ≥7 stars indicated high-quality studies, 4–6 stars as moderate and ≤ 3 stars as low quality [3–5] (Additional file 2).

## Results

### Studies identified

**Fig. 1:**
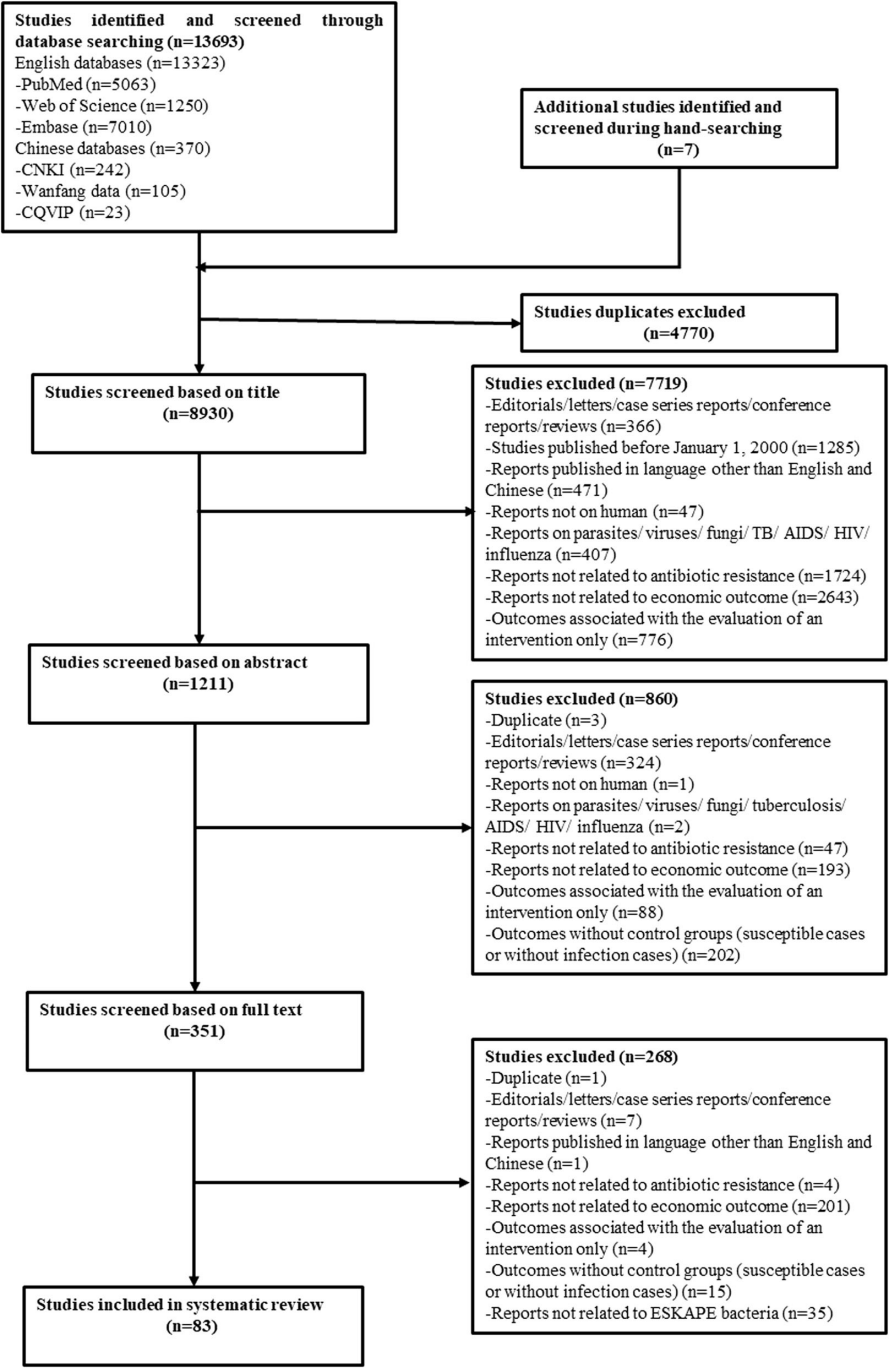
Flowchart of literature search. *CNKI* China National Knowledge Infrastructure, *CQVIP* Chongqing VIP, *AIDS* acquired immunodefiency syndrome, *HIV* human immunodefienccy virus, *ESKAPE Enterococcus spp*, *Staphylococcus aureus*, *Klebsiella pneumoniae*, *Acientobacter baumannii*, *Pseudomonas aeruginosa*, and *Escherichia coli*

A total of 13,693 relevant studies were identified by original database searching. Seven additional studies were identified through other sources. Based on review of titles only, 8930 studies were retrieved after excluding duplicates. Abstract screening resulted in 351 papers for detailed full-text assessment based on the same criteria. Eighty-three studies were finally eligible for this systematic review (Fig.1).

### Study characteristics and quality

Of the 83 eligible studies included in our review, ten were prospective observational studies, and 73 were retrospective studies. Twenty-one studies were conducted in multiple hospital settings. The countries with the largest number of studies were the US (*n* = 40), followed by China (*n* = 16; mainland, China: *n* = 12; Taiwan, China: *n* = 4), Germany (*n* = 6), Thailand (*n* = 5), South Korea (*n* = 4), Canada (*n* = 3), Spain (*n* = 3), Australia (*n* = 1), Colombia (*n* = 1), Italy (*n* = 1), Mexico (*n* = 1), the United Kingdom (*n* = 1), and EU and EEA (31 countries) (*n* = 1). The most studied organism was *S. aureus* (*n* = 32), followed by *Enterococcus* (*n* = 16), *A. baumannii* (*n* = 12), *E. coli* (*n* = 11), *E. coli* or/and *K. pneumoniae* (*n* = 8), *P. aeruginosa* (*n* = 8), and *K. pneumoniae* (*n* = 3). Nine studies reported the economic outcome of ABR in ESKAPE organisms in the intensive care unit (ICU). Five studies included only colonized patients, 17 for hospital-acquired infection, and seven for community-acquired infection. For the sources of infection, 21 studies were bacteremia or septicemia or bloodstream infection (BSI), seven were pneumonia, four were urinary tract infection (UTI), two were surgical site infection, two were skin and soft tissue infection, and two were intra-abdominal infection (IAI) (Additional file 3: Table S1–S7).

For the cost, there were 71 studies on total hospital cost or charge, 12 on antibiotic cost, 11 on hospital cost or charge after culture, 4 on ICU cost, 2 on hospital cost or charge before culture, and 2 on total direct and indirect cost. Most of studies reported that ABR or MDR ESKAPE organisms were significantly associated with higher hospital costs than those with susceptible organisms or those without infection or colonization. Nonetheless, there were no differences in a few studies between the two groups on total hospital cost or charge (16 studies), antibiotic cost (one study), hospital cost before culture (one study), hospital cost after culture (one study). Even, one reported that costs associated with MSSA infection were higher than the costs for similar MRSA cases [26] (Tables 1, 2, 3, 4, 5, 6 and 7). Propensity score matching, simply matching, multivariate linear regression model, generalized linear model, and significant test were the most utilized methods (Additional file 3: Table S1–S7). We judged 66 were high quality studies and 17 were of moderate quality (Additional file 2).

**Table 1:** Studies describing hospital costs among patients with resistant and multi-drug resistant *Staphylococcus aureus*

Author	Bacteria	Comparison		Description of cost	Median cost in 2015 USD						Mean cost in 2015 USD					
		Case	Control		Median cost	Case	Control	Increase	Ratio	*P*-value	Mean cost	Case	Control	Increase	Ratio	*P*-value
Chen et al. [27]	*S. aureus*	MRSA	MSSA	Total hospital cost	Median (IQR)	23,933(14148–34,484)	19,905(10916–39,283)			0.3950						
	S. aureus	MRSA	MSSA	Total hospital cost	Median (IQR)	19,718(13157–28,710)	19,538(10916–3941)			0.9350						
Shorr et al. [28]	S. aureus	MRSA	MSSA	Total hospital charge	Median	77,123	78,399			> 0.05	Mean (SD)	108,117(104303)	104,303(100566)			> 0.05
Taneja et al. [29]	S. aureus	MRSA	MSSA	Total hospital charge	Median	79,150	93,164			0.5100	Mean (SD)	129,393(145555)	145,555(187275)			0.5100
Shorr et al. [30]	S. aureus	MRSA	MSSA	Total hospital cost	Median (IQR)	52,492(23643–91,577)	47,066(20024–92,887)			0.3000						
Itani et al. [31]	S. aureus	MRSA	MSSA	Total hospital charge	Median	25,741	24,311			0.4879	Mean (SD)	58,228(96845)	96,845(139202)			0.5921
Li et al. [32]	S. aureus	MRSA	MSSA	Total hospital cost	Median	1977	2183			0.3900	Mean (SD)	5305(8817)	8817(3017)			0.3900
Park et al. [33]	S. aureus	MRSA	MSSA	Total hospital cost							Mean (SD)	10,319(14220)	14,220(9867)			0.6200
Klein et al. [26]	S. aureus	MRSA	MSSA	Total hospital cost							Mean (95% CI)	34,561(32951–36,170)	32,951(32379–36,038)			0.6900
	S. aureus	MRSA	MSSA	Total hospital cost						0.0450	Mean (95% CI)	38,600(36848–40,766)	36,848(38756–42,776)	− 2166(− 1908-(−2010))	0.95	0.0450
	S. aureus	MRSA	MSSA	Total hospital cost						< 0.001	Mean (95% CI)	14,807(14397–15,216)	14,397(15094–16,093)	− 787(− 697-(− 877))	0.95	< 0.001
Branch-Elliman et al. [34]	S. aureus	MRSA	MSSA	Total hospital cost							Mean (95% CI)					0.4500
Kopp et al. [35]	S. aureus	MRSA	MSSA	Total hospital cost	Median (IQR)	22,799(10007–122,637)	17,692(7279–50,166)			0.1100						
	S. aureus	MRSA	MSSA	Total hospital charge	Median (IQR)	68,857(30536–296,770)	55,161(20323–154,440)			0.1620						
de Kraker et al. [36]	S. aureus	MRSA	MSSA	Excess total hospital cost							Mean (95% CI)	2481(1348–3826)	1348(1933–3110)			*P* > 0.05
Ott et al. [37]	S. aureus	MRSA	MSSA	Total hospital cost	Median (IQR)	94,921(36175–146,202)	60,583(24034–74,790)	34,339(12141–71,412)	1.57	0.0110						
Ben-David et al. [38]	S. aureus	MRSA	MSSA	Total hospital cost	Median (IQR)	146,716(63094–70,878)	54,300(41737–96,367)	92,416(21357–25,490)	2.70	< 0.001						
	S. aureus	MRSA	MSSA	Total hospital cost	Median (IQR)	68,826(42455–108,316)	45,272(23634–65,588)	23,554(18821–42,728)	1.52	0.0050						
	S. aureus	MRSA	MSSA	Hospital cost after culture	Median (IQR)	66,356(31617–134,664)	22,684(13180–54,274)	43,671(18437–80,389)	2.93	< 0.001						
	S. aureus	MRSA	MSSA	Hospital cost after culture	Median (IQR)	30,528(17455–55,896)	23,392(14293–42,786)			0.3000						
McHugh et al. [39]	S. aureus	MRSA	MSSA	Total hospital charge							Mean	65,320	13,797	51,523	4.73	0.0003
Thampi et al. [40]	S. aureus	MRSA	MSSA	Total hospital charge	Median (IQR)	19,743(9490–42,061)	12,195(7116–29,294)	7547(2374–12,767)	1.62	0.0294						
Rubio-Terres et al. [41]	S. aureus	MRSA	MSSA	Total hospital cost							Mean	16,669	14,850	1819	1.12	< 0.05
Reed et al. [42]	S. aureus	MRSA	MSSA	Total hospital cost	Median (range)	34,763(17499–60,252)	20,103(11995–26,845)	14,660(5505–33,407)	1.73	0.0001	Mean (SD)	43,715(33886)	33,886(24001)	18,544(9885)	1.74	0.0001
Engemann et al. [43]	S. aureus	MRSA	MSSA	Total hospital charge	Median (IQR)	127,047(55293–187,729)	72,615(39992–126,279)	54,432(15301–61,450)	1.75	< 0.001						
	S. aureus	MRSA	Without infection	Total hospital charge	Median (IQR)	127,047(55293–187,729)	40,516(21509–57,447)	86,531(33784–130,282)	3.14	< 0.001						
	S. aureus	MRSA	MSSA	Total hospital charge	Median (IQR)					0.0300						
Anderson et al. [44]	S. aureus	MRSA	Without infection	Total hospital charge	Median (IQR)	101,841(49115–164,750)	49,916(22878–78,128)	51,925(26237–86,622)	2.04	< 0.0001						
	S. aureus	MRSA	MSSA	Total hospital charge	Median (IQR)	101,841(49115–164,750)	71,736(28610–111,800)	30,106(20505–52,950)	1.42	0.0010						
Song et al. [45]	S. aureus	MRSA	MSSA	Excess total hospital charge	Median (95% CI)			56,900(53137–60,662)		< 0.001						
	S. aureus	MRSA	MSSA	Excess total hospital charge	Median (95% CI)			180,948(174793–187,102)		< 0.001						
Filice et al. [46]	S. aureus	MRSA	MSSA	Total hospital cost	Median (range)	30,027(5178–99,399)	7712(0–40,102)	22,315(5178–59,297)	3.89	< 0.001						
	S. aureus	MRSA	MSSA	Antibiotic cost	Median (range)	162(7–581)	24(0–385)	138(7–195)	6.76	< 0.001						
Nelson et al. [47]	S. aureus	MRSA	MSSA	Total hospital cost							Mean	38,294	24,127	14,167	1.59	< 0.0001
Lee et al. [48]	S. aureus	MRSA	MSSA	Total hospital cost	Median	33,717	5392	28,325	6.25	< 0.001	Mean	34,582	9245	25,337	3.74	< 0.001
	S. aureus	MRSA	MSSA	ICU cost	Median	27,349	4074	23,275	6.71	< 0.001	Mean	27,680	7187	20,493	3.85	< 0.001
Resch et al. [49]	S. aureus	MRSA	MSSA	Total hospital cost							Mean	24,964	12,191	12,772(30229)	2.05	< 0.000
	S. aureus	MRSA	MSSA	Total hospital cost							Mean	21,440	10,134	11,306(27376)	2.12	< 0.000
Nelson et al. [50]	S. aureus	MRSA	MSSA	Excess total hospital cost							Mean (95% CI)			34,315(32689–35,942)		< 0.0001
	S. aureus	MRSA	MSSA	Excess total hospital cost							Mean (95% CI)			26,103(11828–40,379)		< 0.0001
	S. aureus	MRSA	MSSA	Excess total hospital cost							Mean (95% CI)			29,190(24547–33,833)		< 0.0001
Xu et al. [51]	S. aureus	MRSA	MSSA	Total hospital cost							Mean (SD)	4139(5032)	5032(3529)	1784(1503)	1.76	0.0060
	S. aureus	MRSA	MSSA	Antibiotic cost							Mean (SD)	223(199)	199(173)	82(26)	1.58	0.0070
Capitano et al. [52]	S. aureus	MRSA	MSSA	Total infection cost	Median (range)	3490(1137–11,908)	1783(359–9726)	1707(778–2182)	1.96	< 0.001						
Cosgrove et al. [53]	S. aureus	MRSA	MSSA	Hospital charge after culture	Median (IQR)	36,347(19265–69,442)	26,426(13754–50,272)	9920(5512–19,169)	1.38	0.0080						
	S. aureus	MRSA	MSSA	Hospital cost after culture	Median (IQR)	20,158(10685–38,512)	14,656(7627–27,882)	5502(3058–10,630)	1.38	0.0080						
	S. aureus	MRSA	MSSA	Hospital charge before culture	Median (IQR)	2868(0–37,301)	1382(0–23,414)	1486(0–13,887)	2.07	0.0400						
Lodise et al. [54]	S. aureus	MRSA	MSSA	Hospital cost after culture							Mean	30,435	15,000	15,435	2.03	< 0.001
	S. aureus	MRSA	MSSA	Hospital cost after culture							Mean (95% CI)	28,885(22839–36,533)	22,839(12784–19,040)	13,265(10055–17,493)	1.85	0.0010
Kim et al. [55]	S. aureus	MRSA	Without infection	Total hospital cost	Median (IQR)	20,356(10381–315,530)	8462(5267–18,686)	11,895(5115–296,844)	2.41	< 0.05						
	S. aureus	MRSA	Without infection	Total hospital charge	Median (IQR)	4911(2360–8387)	2486(917–4350)	2426(1444–4037)	1.98	< 0.05						
Fu et al. [56]	S. aureus	MRSA	Without infection	Total hospital cost	Median (Q)	15,763(160950)	2185(43820)	13,578(117130)	7.21	0.0010						
Engler Husch et al. [57]	S. aureus	MRSA	Without colonizaiton	Excess total hospital cost							Mean (95% CI)			2193(1699–2688)		< 0.01

*MRSA* methicillin resistant *S. aureus*, *MSSA* methicillin susceptible *S. aureus*, *ICU* intensive care unit, *IQR* interquartile range, *Q* quartile, *USD* the United States Dollars, *SD* standard deviation, *CI* confidence interval

**Table 2:** Studies describing hospital costs among patients with resistant and multi-drug resistant *Enterococcus*

Author	Bacteria	Comparison		Description of cost	Median cost in 2015 USD						Mean cost in 2015 USD					
		Case	Control		Median cost	Case	Control	Increase	Ratio	*P*-value	Mean cost	Case	Control	Increase	Ratio	*P*-value
Butler et al. [58]	Enterococcus	VRE	VSE	Total hospital cost	Median (IQR)	48,121(18640–107,280)	23,880(1443–47,862)	24,241(17197–59,418)	2.02	< 0.001						
	Enterococcus	VRE	Without infection	Total hospital cost	Median (IQR)	48,121(18640–107,280)	9362(6417–15,423)	38,759(12223–91,857)	5.14	< 0.001						
Ford et al. [59]	Enterococcus	VRE	VSE	Total hospital cost	Median	177,503	88,751	88,751	2.00	0.0003						
Kramer et al. [60]	*E. faecium*	VRE	VSE	Total hospital cost	Median (IQR)	89,241(53110–174,619)	56,967(24993–132,854)	32,274(28117–41,765)	1.57	< 0.000						
Cheah et al. [61]	Enterococcus	VRE	VSE	Total hospital cost	Median (IQR)	86,286(47297–170,205)	43,051(27497–99,883)	43,235(19800–70,322)	2.00	0.0020						
Lloyd-Smith et al. [62]	Enterococcus	VRE	VSE	Total hospital cost							Mean (SD)	46,924(55881)	55,881(17783)	33,855(38098)	3.59	< 0.0001
	Enterococcus	VRE	VSE	Excess total hospital cost							Mean (95% CI)					> 0.05
Adams et al. [63]	Enterococcus	VRE	VSE	Total hospital cost							Mean (95% CI)	51,020(43463–59,891)	43,463(37797–48,434)	8233(5666–11,457)	1.19	0.0040
Gearhart et al. [64]	Enterococcus	VRE	VSE	Total hospital cost							Mean	81,106	36,705	44,401	2.21	< 0.07
Webb et al. [65]	E. faecium	VRE	VSE	Total hospital cost							Mean	158,795	97,472	61,323	1.63	< 0.05
Carmeli et al. [66]	Enterococcus	VRE	VSE	Total hospital charge							Mean	77,473	47,142	30,331	1.64	< 0.001
Nguyen et al. [67]	Enterococcus	VRE	VSE	Total hospital charge							Mean	77,084	26,600	50,484	2.90	< 0.0001
Jung et al. [68]	Enterococcus	VRE	VSE	Total hospital cost							Mean	11,741	9877	1864	1.19	0.2680
	Enterococcus	VRE	VSE	ICU cost							Mean	6071	5303	768	1.14	0.0290
Puchter et al. [69]	Enterococcus	VRE	VSE	Total hospital cost	Median (IQR)	93,044(55258–200,170)	61,858(33672–123,176)	31,186(21587–76,994)	1.50	0.0300						
	Enterococcus	VRE	VSE	Hospital cost before culture	Median (IQR)	28,866(17204–51,932)	26,780(11894–39,884)			0.3860						
	Enterococcus	VRE	VSE	Hospital cost after culture	Median (IQR)	61,257(28693–130,128)	19,399(16652–65,441)	41,857(12041–64,686)	3.16	0.0490						
Jiang et al. [70]	Enterococcus	VRE	VSE	Antibiotic cost	Median	3406	1031	2375	3.30	< 0.0001	Mean (SD)	5009(3361)	3361(1612)	3071(1749)	2.58	< 0.0001
Pelz et al. [71]	Enterococcus	VRE	Without infection	ICU cost	Median	50,187	28,494	21,693	1.76	0.0300						
Song et al. [72]	Enterococcus	VRE	Without infection	Total hospital charge	Median	170,917	64,235	106,682	2.66	< 0.05						
Engler Husch et al. [57]	Enterococcus	VRE	Without colonizaiton	Excess total hospital cost							Mean (95% CI)			1167(802–1533)		< 0.01

*VRE* vancomycin resistant *Enterococcus*, *VSE* vancomycin susceptible *Enterococcus*, *ICU* intensive care unit, *IQR* interquartile range, *USD* the United States Dollars, *SD* standard deviation, *CI* confidence interval

**Table 3:** Studies describing hospital costs among patients with resistant and multi-drug resistant *E. coli and Klebsiella spp. / K. pneumoniae*

Author	Bacteria	Comparison		Description of cost	Median cost in 2015 USD						Mean cost in 2015 USD					
		Case	Control		Median cost	Case	Control	Increase	Ratio	*P*-value	Mean cost	Case	Control	Increase	Ratio	*P*-value
Maslikowska et al. [73]	*E. coli* and Klebsiella spp.	ESBL positive	ESBL negative	Total hospital cost (direct and indirect)	Median (IQR)	10,379(1245–41,644)	7786(436–58,035)	2593(80916391)	1.33	0.0391						
	E. coli and Klebsiella spp.	ESBL positive	ESBL negative	Total hospital cost	Median (IQR)	2451(285–101,668)	1868(98–10,994)	583(187–90,675)	1.31	0.0436						
Hu et al. [74]	E. coli and Klebsiella spp.	ESBL positive	ESBL negative	Total hospital cost							Mean	4119	2308	1811	1.78	< 0.001
MacVane et al. [75]	E. coli and Klebsiella spp.	ESBL positive	ESBL negative	Total hospital cost	Median (IQR)	11,085(7065–16,325)	7310(5848–12,025)	3775(1217–4300)	1.52	0.0200						
	E. coli and Klebsiella spp.	ESBL positive	ESBL negative	Antibiotic cost	Median (IQR)	55(35–82)	7(6–12)	48(29–69)	7.58	< 0.001						
Yang et al. [76]	E. coli and *K. pneumoniae*	ESBL positive	ESBL negative	Antibiotic cost							Mean (SD)	677(466)	466(296)	399(170)	2.43	0.0140
Apisarnthanarak et al. [77]	E. coli and K. pneumoniae	ESBL positive	ESBL negative	Hospital cost after culture	Median (range)	703(49–3626)	245(61–2127)	458(−11–1499)	2.87	< 0.001						
Lautenbach et al. [78]	E. coli and K. pneumoniae	ESBL positive	ESBL negative	Hospital charge after culture	Median	96,788	32,313	64,475	3.00	< 0.001						
Lee et al. [79]	E. coli and Klebsiella spp.	ESBL positive	ESBL negative	Total infection cost							Mean (SD)	51,886(49695)	49,695(20811)	20,641(28885)	1.66	0.0380
Apisarnthanarak et al. [80]	E. coli and K. pneumoniae	ESBL positive	ESBL negative	Total hospital cost	Median (range)	2428(53–5300)	1408(38–4734)	1020(15–566)	1.72	< 0.05						
	E. coli and K. pneumoniae	ESBL positive	Without infection	Total hospital cost	Median (range)	2428(53–5300)	684(53–2725)	1744(0–2575)	3.55	< 0.05						

*ESBL* extended-spectrum β-lactamases, *IQR* interquartile range, *USD* the United States Dollars, *SD* standard deviation

**Table 4:** Studies describing hospital costs among patients with resistant and multi-drug resistant *E. coli*

Author	Bacteria	Comparison		Description of cost	Median cost in 2015 USD						Mean cost in 2015 USD					
		Case	Control		Median cost	Case	Control	Increase	Ratio	*P*-value	Mean cost	Case	Control	Increase	Ratio	*P*-value
de Kraker et al. [36]	E. coli	Third generation cephalosporin resistant E. coli	Third generation cephalosporin susceptible E. coli	Excess total hospital cost							Mean (95% CI)	1861(776–3319)	776(530–1236)	985(246–2084)	2.12	< 0.05
Tumbarello et al. [81]	E. coli	ESBL positive	ESBL negative	Total hospital cost							Mean (SD)	13,709(16312)	16,312(6683)	5026(9629)	1.58	0.0300
Thaden et al. [82]	E. coli	MDR	Non-MDR	Total hospital cost	Median (IQR)	9527(5915–19,648)	7503(4301–13,447)	2024(1614–6201)	1.27	< 0.0001	Mean (SD)	18,917(29394)	29,394(24416)	4141(4978)	1.28	< 0.0001
Apisarnthanarak et al. [83]	E. coli	ESBL positive	ESBL negative	Hospital cost after culture	Median (range)	662(54–3981)	243(66–2335)	419(− 13–1646)	2.72	< 0.05						
Apisarnthanarak et al. [84]	E. coli	ESBL positive	Without infection	Total hospital cost	Median (range)	662(54–3981)	136(29–991)	527(25–2990)	4.89	< 0.001						
Alam et al. [85]	E. coli	Resistant to at least one antibiotic	Sensitive to all six antibiotics	Total hospital cost							Mean (SD)	33(46)	46(42)	8(4)	1.33	0.0060
	E. coli	Resistant to at least one antibiotic	Sensitive to all six antibiotics	Antibiotic cost							Mean (SD)	7(14)	14(3)	3(11)	1.72	< 0.001
Esteve-Palau et al. [86]	E. coli	ESBL positive	ESBL negative	Total hospital cost	Median (IQR)	4877(3537–9645)	3379(2318–5832)	1498(1220–3813)	1.44	0.0070						
	E. coli	ESBL positive	ESBL negative	Antibiotic cost	Median (IQR)	259(53–605)	22(8–82)	238(45–523)	12.00	< 0.001						
Cornejo-Juarez et al. [87]	E. coli	ESBL positive	ESBL negative	Total hospital cost							Mean (SD)	6535(4352)	4352(3176)	1808(1176)	1.38	0.0100
	E. coli	ESBL positive	ESBL negative	Antibiotic cost							Mean (SD)	2801(2275)	2275(1669)	848(607)	1.43	0.0200
Xu et al. [51]	E. coli	MDR	Non-MDR	Total hospital cost							Mean (SD)	3645(4948)	4948(3068)	1574(1881)	1.76	< 0.001
	E. coli	MDR	Non-MDR	Antibiotic cost							Mean (SD)	234(301)	301(179)	81(122)	1.52	< 0.001
Meng et al. [88]	E. coli	CREC	CSEC	Total hospital cost	Median	12,670	10,290			> 0.05						
	E. coli	CREC	Without infection	Total hospital cost	Median	12,670	2818	9851	4.50	< 0.000						
Leistner et al. [89]	E. coli	ESBL positive	ESBL negative	Total hospital cost	Median (IQR)	21,712(9016–59,726)	23,841(8060–67,701)			0.3590						

*ESBL* extended-spectrum β-lactamases, *MDR* multi-drug resistance, *CREC* carbapenem resistace *E. coli*, *CSEC* carbapenem susceptible *E. coli*, *IQR* interquartile range, *USD* the United States Dollars, *SD* standard deviation, *CI* confidence interval

**Table 5:** Studies describing hospital costs among patients with resistant and multi-drug resistant *K. pneumoniae*

Author	Bacteria	Comparison		Description of cost	Median cost in 2015 USD						Mean cost in 2015 USD					
		Case	Control		Median cost	Case	Control	Increase	Ratio	*P*-value	Mean cost	Case	Control	Increase	Ratio	*P*-value
Thaden et al. [82]	K. pneumoniae	MDR	Non-MDR	Total hospital cost	Median (IQR)	46,934(12470–153,881)	11,183(5955–29,452)	35,751(6515–124,429)	4.20	0.0300	Mean (SD)	115,868(163881)	163,881(48116)	86,991(115765)	4.01	0.0300
Xu et al. [51]	K. pneumoniae	MDR	Non-MDR	Total hospital cost							Mean (SD)	5132(10165)	10,165(4603)	1954(5563)	1.61	0.0010
	K. pneumoniae	MDR	non-MDR	Antibiotic cost							Mean (SD)	263(378)	378(361)			0.5900
Huang et al. [90]	K. pneumoniae	CRKP	CSKP	Total hospital cost	Median (range)	22,207(10938–41,559)	11,368(4730–24,634)	10,838(6208–16,925)	1.95	< 0.001						
	K. pneumoniae	CRKP	CSKP	Hospital cost after culture	Median (range)	8912(3248–20,173)	6677(2554–14,832)	2235(693–5341)	1.33	0.0030						
	K. pneumoniae	CRKP	CSKP	Antibitoic cost	Median (range)	2139(710–4926)	933(240–2468)	1206(470–2457)	2.29	< 0.001						

*MDR* multi-drug resistance, *CRKP* carbapenem resistace *K. pneumoniae*, *CSEC* carbapenem susceptible *K. pneumoniae*, *IQR* interquartile range, *USD* the United States Dollars, *SD* standard deviation

**Table 6:** Studies describing hospital costs among patients with resistant and multi-drug resistant *P. aeruginosa*

Author	Bacteria	Comparison		Description of cost	Median cost in 2015 USD						Mean cost in 2015 USD					
		Case	Control		Median cost	Case	Control	Increase	Ratio	*P*-value	Mean cost	Case	Control	Increase	Ratio	*P*-value
Chen et al. [91]	*P. aeruginosa*	CRPA	CSPA	Total hospital cost	Median (IQR)	6122(3230–13,779)	3191(1860–7313)	2932(1370–6466)	1.92	< 0.001						
	P. aeruginosa	CRPA	CSPA	Total hospital cost	Median (IQR)	5743(3147–11,957)	4678(2602–9596)	1065(545–2361)	1.23	0.0150						
Lautenbach et al. [92]	P. aeruginosa	IRPA	ISPA	Total hospital cost	Median (IQR)	111,871(39270–313,857)	66,549(26338–180,391)	45,322(12931–133,466)	1.68	< 0.001						
Gasink et al. [93]	P. aeruginosa	Fluoroquinolones resistant P. aeruginosa	Fluoroquinolones susceptible P. aeruginosa	Total hospital charge	Median (IQR)	85,729(30439–259,944)	67,033(25805–171,704)	18,696(4634–88,239)	1.28	0.0080						
Morales et al. [94]	P. aeruginosa	MDR	Non-resistant	Total hospital cost	Median (IQR)	9810(7033–15,036)	4132(2987–5058)	5678(4046–9978)	2.37	< 0.001	Mean (95% CI)	22,503(17445–27,560)	17,445(5765–8780)	15,231(11680–18,780)	3.09	< 0.001
	P. aeruginosa	Resistant	Non-resistant	Total hospital cost	Median (IQR)	8961(7119–11,865)	4132(2987–5058)	4829(4132–6808)	2.17	< 0.001	Mean (95% CI)	18,207(13058–23,358)	13,058(5765–8780)	10,935(7293–14,578)	2.50	< 0.000
Xu et al. [51]	P. aeruginosa	MDR	Non-MDR	Total hospital cost							Mean (SD)	13,820(9536)	9536(5450)	9973(4087)	3.59	< 0.001
	P. aeruginosa	MDR	Non-MDR	Antibiotic cost							Mean (SD)	884(321)	321(322)	559(−1)	2.72	< 0.001
Lautenbach et al. [95]	P. aeruginosa	IRPA	ISPA	Hospital cost after culture							Mean (95% CI)	295,529(241780–349,278)	241,780(177913–212,678)	100,234(63867–136,600)	1.51	< 0.001
Gasink et al. [96]	P. aeruginosa	Aztreonam resistant P. aeruginosa	Aztreonam susceptible P. aeruginosa	Hospital charge after culture	Median	94,483	65,131	29,352	1.45	0.0100						
Eagye et al. [97]	P. aeruginosa	MRPA	MSPA	Total cost (direct and indirect)	Median (IQR)	103,907(28591–188,951)	33,631(13529–103,906)	70,276(15062–85,045)	3.09	< 0.001						
	P. aeruginosa	MRPA	Without infection	Total cost (direct and indirect)	Median (IQR)	103,907(28591–188,951)	26,563(18012–41,908)	77,344(10579–147,042)	3.91	< 0.001						

*CRPA* carbapenem resitance *P. aeruginosa*, *CSPA* carbapenem susceptible *P. aeruginosa*, *IRPA* imipenem resitance *P. aeruginosa*, *ISPA* imipenem susceptible *P. aeruginosa*, *MRPA* meropenem resitance *P. aeruginosa*, *MSPA* meropenem susceptible *P. aeruginosa*, *MDR* multi-drug resistance, *IQR* interquartile range, *USD* the United States Dollars, *SD* standard deviation, *CI* confidence interval

**Table 7:** Studies describing hospital costs among patients with resistant and multi-drug resistant *A. baumannii*

Author	Bacteria	Comparison		Description of cost	Median cost in 2015 USD						Mean cost in 2015 USD					
		Case	Control		Median cost	Case	Control	Increase	Ratio	*P*-value	Mean cost	Case	Control	Increase	Ratio	*P*-value
Cui et al. [98]	A. baumannii	IRAB	ISAB	Total hospital cost	Median (IQR)	16,835(7619–46,479)	7783(3583–36,235)	9052(4035–10,244)	2.16	< 0.01						
	A. baumannii	IRAB	ISAB	Antibiotic cost	Median (IQR)	2558(793–5799)	1257(519–3835)	1301(274–1964)	2.03	< 0.01						
Zhen et al. [99]	A. baumannii	CRAB	CSAB	Total hospital cost							Mean (SD)	30,575(17)	17(11)	10,792(7)	1.55	< 0.000
	A. baumannii	CRAB	CSAB	Antibiotic cost							Mean (SD)	3047(19)	19(11)	1355(8)	1.80	< 0.000
Lemos et al. [108]	A. baumannii	CRAB	CSAB	Total hospital cost							Mean (95%CI)	11,969(10593–13,154)	10,593(6540–8452)	4541(2970–5948)	1.61	< 0.001
	A. baumannii	CRAB	CSAB	Antibiotic cost							Mean (95%CI)	3854(3234–4383)	3234(2117–3238)	1198(407–1996)	1.45	0.0020
	A. baumannii	CRAB	CSAB	Total hospital cost							Mean (SD)	12,457(7728)	7728(4150)	4894(3579)	1.65	< 0.001
	A. baumannii	CRAB	CSAB	Antibiotic cost							Mean (SD)	4316(4242)	4242(2152)	1708(2091)	1.65	0.0020
Lautenbach et al. [100]	A. baumannii	IRAB	ISAB	Hospital charge after culture							Mean (95% CI)	393,086(276636–509,536)	276,636(252785–396,002)	68,692(23851–113,533)	1.21	0.0300
Lee et al. [101]	A. baumannii	MDR	Non-MDR	Total hospital cost							Mean	15,881	7739	8141	2.05	0.0460
	A. baumannii	MDR	Non-MDR	ICU cost							Mean	13,933	4311	9622	3.23	0.0020
Wu et al. [102]	A. baumannii	MDR	Non-MDR	Total hospital cost	Median (Q)	24,897(156940)	8823(63540)	16,074(93390)	2.82	< 0.01						
Guo et al. [103]	A. baumannii	MDR	Non-MDR	Total hospital cost	Median (IQR)	10,452(4742–21,484)	3759(1586–9310)	6693(3156–12,174)	2.78	< 0.001	Mean (SD)	14,087(12302)	12,302(11045)	6638(1257)	1.89	< 0.001
Xu et al. [51]	A. baumannii	MDR	Non-MDR	Total hospital cost							Mean (SD)	5446(6481)	6481(3602)	2347(2879)	1.76	0.0250
	A. baumannii	MDR	Non-MDR	Antibiotic cost							Mean (SD)	222(267)	267(166)			0.0540
Lee et al. [104]	A. baumannii	MDR	Non-MDR	Total hospital cost							Mean (SD)	12,515(8465)	8465(5375)	6003(3090)	1.92	0.0010
	A. baumannii	MDR	Non-MDR	Antibiotic cost							Mean (SD)	3021(1822)	1822(1760)	866(62)	1.40	0.0140
Thatrimontrichai et al. [105]	A. baumannii	CRAB	CSAB	Total hospital cost	Median (range)	11,785(2176–45,664)	9745(5266–24,170)			> 0.05						
	A. baumannii	CRAB	Without infection	Total hospital cost	Median (range)	11,785(2176–45,664)	7806(3877–33,608)			> 0.05						
Young et al. [106]	A. baumannii	MDR	Without infection	Total hospital charge							Mean	372,424	165,032	207,392	2.26	< 0.001
	A. baumannii	MDR	Without infection	Total hospital charge							Mean			73,924		< 0.05
Wilson et al. [107]	A. baumannii	MDR	Without infection	Total hospital cost							Mean (SD)	269,823(215236)	215,236(156080)	131,961(59155)	1.96	< 0.01

*CRAB* carbapenem resitance *A. baumannii*, *CSAB* carbapenem susceptible *A. baumannii*, *IRAB* imipenem resitance *A. baumannii*, *ISAB* imipenem susceptible *A. baumannii*, *MDR* multi-drug resistance, *IQR* interquartile range, *Q* quartile, *USD* the United States Dollars, *SD* standard deviation, *CI* confidence interval

#### *Staphylococcus aureus*

For *S. aureus*, the control groups were categorized into two groups namely methicillin susceptible *S. aureus* (MSSA) and non-infection. Mean or median total hospital cost or charge among inpatients with MRSA was 1.12 times-6.25 times higher than that for MSSA hospitalizations [37–47, 51]. The median cost difference ranged from $7547 in Canada [40] to $180,948 in the US [45], and the mean cost difference ranged from $1784 in China [51] to $51,523 in the US [39]. Median cost in total infection cost, ICU cost, and hospital charge before culture among inpatients with MRSA were 1.96 times [52], 6.71 times [48], and 2.07 times [53] as high as that for MSSA cases, respectively. Compared with inpatients with MSSA, MRSA cases were associated with ranging from 1.58 times [51] to 6.75 times [46] of mean or median antibiotic cost, and ranging from 1.38 times [53] to 2.93 times [38] of mean or median hospital cost or charge after culture. However, there were no differences in total hospital cost or charge in 11 studies [26–35, 60] and in hospital cost after culture in one study [38] between the two groups.

In addition, compared with inpatients without infection, those with MRSA were associated with 1.98 times-7.21 times higher total hospital cost or charge [44, 55, 56]. ICU is a significant driver behind increased hospital costs, MRSA in the ICU was associated with four times higher additional total hospital cost than that in general ward [38]. Type of infection is also an important factor of hospital costs [38]. Compared with inpatients with MSSA BSI, surgical site infection, and pneumonia, those with MRSA were significantly associated with 52–170% [38–42, 55], 75–214% [43, 44, 68], and 57% [37] of increased median total hospital cost or charge, respectively. 12–373% [39, 41, 42] and 78% [74] of increased mean total hospital cost or charge were incurred among inpatients with MRSA BSI and IAI than those with MSSA, respectively. Nevertheless, it is showed that there were no differences in total hospital cost or charge among BSI [26, 33, 36], breast abscess [34], skin and soft tissue infection [31, 32, 34, 72], and pneumonia [27–30] between the two groups. There was an additional median total hospital charge of $ 56,900 [45] and mean total hospital cost of $ 2193 [57] for MRSA colonization than that for MSSA colonization and those without colonization, respectively.

Contrary to the historical studies at the hospital level, Klein et al. found that costs associated with MSSA-related infection were similar with and even surpass costs of MRSA-related infections [26] (Table 1 and Additional file 3: Table S1 and S8 ).

#### *Enterococcus*

For *Enterococcus*, the control groups were also divided into two groups namely vancomycin susceptible *Enterococcus* (VSE) and non-infection. Median total hospital cost among inpatients with VRE BSI was 1.57–2.02 times higher than that for VSE cases [58–61], with the increased cost ranging from $24,241 [58] and $88,751 in the US [59]. In addition, inpatients with VRE were associated with 19–259% of increased mean total hospital cost or charge than those with VSE [62–67], ranging from $8233 [63] to $61,323 [65]. However, some studies reported that there were no differences in total hospital cost [62, 68] or hospital cost before culture [69].

Median costs in antibiotic cost and hospital cost after culture among inpatients with VRE were 3.30 times [70] and 3.16 times [69] as high as that for VSE cases, respectively. VRE colonization was found not to be a significant factor associated with total hospital cost, nonetheless, it resulted in a significant increase in ICU cost after controlling confounding factors using propensity score matching [68].

Compared with inpatients without infection, those with VRE were associated with 1.76 times higher ICU cost [71], and VRE BSI cases were associated with 2.66 times [72] higher total hospital charge and 5.14 times higher total hospital cost [58]. We also found that VRE colonized inpatients led to a significant cost increase of $1167 than those without colonization after controlling for variables [57] (Table 2 and Additional file 3: Table S2 and S8).

#### *Klebsiella pneumoniae* and *Escherichia coli*

For both *K. pneumoniae* and *E. coli*, cases with extended spectrum β-lactamases (ESBL)-positive cultures were associated with significantly higher hospital costs or charges compared with ESBL-negative cases [73–75, 80] or those without infection [80]. Direct and indirect cost and total hospital cost for ESBL-positive inpatients was 1.33 times [73] and 1.31–1.72 times [73, 80] as much as that for ESBL-negative cases, respectively. ESBL-positive IAI can attribute to 78% of increased total hospital cost than ESBL-negative cases [74]. UTI with ESBL-producing bacteria was significantly associated with a 1.52-fold increase in median hospital cost, 7.58-fold increase in median antibiotic cost [75], and 2.43-fold increase in mean antibiotic cost [76] compared to non-ESBL-producing organisms. 187–200% of additional hospital cost or charge after culture [77, 78] and 66% of additional total infection cost [79] attributable to ESBL-producing were found [79]. In addition, compared with inpatients without infection, ESBL-positive cases were associated with 3.55-fold total hospital cost [80] (Table 3 and Additional file 3: Table S3 and S8).

For *E. coli* only, BSI due to third-generation cephalosporins resistance, ESBL-positive, and MDR was associated with 2.12 times, 1.58 times, and 1.28 times of total hospital cost than that for third-generation cephalosporins susceptibility, ESBL-negative, and non-MDR, respectively [36, 82, 90]. Two studies from Thailand explored that community-acquired infection due to ESBL-producing increased hospital costs, with 2.72 times hospital cost after culture than that for non-ESBL-producing, and 4.89 times total hospital cost than that for non-infection [81, 83]. The total hospital cost and antibiotic cost of UTI due to resistance to at least one antibiotic (ampicillin, trimethoprim, amoxicillin/clavulanic acid, cephalexin, ciprofloxacin or nitrofurantoin) and ESBL-producing were considerably higher than that for sensitive to all six antibiotics and non-ESBL-producing after accounting for confounding factors, respectively [84, 85]. Similarly, ESBL-positive colonization was significantly associated with higher total hospital cost and antibiotic cost, with a mean difference of $1808 and $848, respectively [86]. In addition, the mean difference in total hospital cost and antibiotic cost was $1574 and $81 between the MDR and non-MDR group in univariate analyses [51]. However, one study reported that there was no significant difference in total hospital cost between ESBL-positive and ESBL-negative BSI after matching for confounders [87] (Table 4 and Additional file 3: Table S4 and S8).

For *K. pneumoniae* only, adjusted median total hospital cost for inpatients with MDR *K. pneumoniae* bloodstream infection was 4.20 time higher than that for non-MDR cases in US [90]. Median costs in total hospital, hospital cost, hospital cost after culture, and antibiotic cost for carbapenem resistant *K. pneumoniae* (CRKP) cases were 1.95 times, 1.33 times, and 2.29 times as high as that for carbapenem susceptible *K. pneumoniae* cases, respectively [89]. One study conducted in China found that the difference in total hospital cost between MDR and non-MDR group was significant [89], while there was no difference in antibiotic cost in a univariate analysis [51] (Table 5 and Additional file 3: Table S5 and S8).

#### *Pseudomonas aeruginosa*

For *P. aeruginosa*, median total hospital cost for inpatients with CRPA was 1.23 times − 1.68 times higher than that for those with CSPA after balancing baseline characteristics [91, 92], ranging from $1065 in China [91] to $45,322 in the US [92]. In univariate analyses, CRPA contributed to 1.51 times of mean hospital cost after culture than CSPA [95], and Eagye et al. found that inpatients with CRPA were associated with 3.09 times median total cost (indirect and direct cost) as high as CSPA cases and 3.91 times higher than those without infection [97]. Resistance and MDR were independently predictive of an increased total hospital cost compared with non-resistance and non-MDR (2.50-fold for resistance vs non-resistance; 3.09-fold for MDR vs non-resistance; 3.59-fold for MDR vs non-MDR) [51, 94]. [97]. Two studies conducted in the same hospital setting in the US found that resistance to fluoroquinolones and resistance to aztreonam are risk factors for increased total hospital charge and hospital charge after culture, respectively, compared with susceptible cases [93, 96] (Table 6 and Additional file 3: Table S6 and S8).

#### *Acinetobacter baumannii*

For *A. baumannii*, two studies indicated that inpatients with CRAB were associated with higher total hospital cost and antibiotic cost than CSAB cases after adjusting some confounding factors [98, 99, 108], and the mean difference in total hospital cost between MDR and non-MDR group ranged from $6693 to $16,074 in China [102, 103]. In univariate analyses, mean hospital charge after culture for CRAB cases was 1.21 times − 1.65 times higher than that for CSAB cases [100, 108], and one study found a significant difference in total hospital cost but not in antibiotic cost among MDR and non-MDR group [51]. Lee et al. explored that MDR colonization was associated with significantly increased ICU cost and total hospital cost [101]. One study in Taiwan, China found significant differences in total hospital cost and antibiotic cost among MDR and non-MDR bacteremia [104]. In addition, MDR inpatients were associated with twice times total hospital charge or cost compared with those without infection [106, 107]. However, there was no significant difference for total hospital cost among infants with ventilator associated pneumonia in the ICU after matching baseline variables between CRAB and CSAB group, and between CRAB and non-infected group [105] (Table 7 and Additional file 3: Table S7 and S8).

## Discussion

ESKAPE species are among the most common bacterial organisms in healthcare-acquired infections, posing a great threat to human health and becoming increasingly more resistant to commonly used antibiotics. This systematic review updates the evidences regarding the economic burden of ABR or MDR ESKAPE organisms compared to susceptible cases or those without infection or colonization. Directing attention towards the ESKAPE organisms can help us to better combat the wide challenge of ABR, especially MDR. The studies on the economic cost of ABR are limited chiefly to high-income countries, even though, the current status of ABR may be more serious in the low- and middle-income countries because of scarcity of new medicines, diagnostic tools, and interventions, thus, the value of the economic burden of ABR might be underestimated.

We find that ABR in ESKAPE organisms, is not always, but usually, associated with significantly higher economic burden. In some studies, there are no significant differences in total hospital cost or charge between MRSA and MSSA group [27–36], VRE and VSE group [62, 68], ESBL-positive and ESBL-negative group [87], and CRAB and CSAB group [105]. In addition, the difference in antibiotic cost between MDR and non-MDR group among *A. baumannii* and *E. coli*, the difference in hospital cost before culture between VRE and VSE [69], and the difference in hospital cost after culture between MRSA and MSSA [38] have not reached statistical significance as well [51]. The above results may be closely related to study design and patient level factors. Patients with ABR, especially MDR are usually more likely to have more severe illness than those with susceptibility or non-infection, and more likely to be admitted in the ICU, be undergone more surgery, and be taken more antibiotic treatment. It is possible that ABR, especially MDR may be associated with higher hospital cost; however, these studies may lack statistical power to detect a significant difference. The results without significant differences are usually drawn after adjustment for confounding variables. If severity of illness is controlled for and all cases are treated similarly, then a cost difference will not be expected. In addition, patients level factors including age (e.g. adult patients, children, or adolescents), source of infection (e.g. BSI, UTI, or IAI), and whether the organism is colonization or infection, are associated with disease status as well, thus may influence the conclusion. Further studies with large sample size, different patients level factors, and controlling confounding factors, are need in the future.

There is one study that even suggested that costs associated with MSSA infection have converged with and may surpass costs of similar MRSA cases [26], which is different compared to historical studies. There are some potential reasons for this diverging result. As mentioned, any study design which controls for severity of illness would be expected to bias the study towards a negative result. It is reported that compared with MSSA, MRSA was associated with a higher mortality rate, thus, we could conclude that it produced a higher severity of illness, which would be expected to require more patient services; however, in this study, patients that died in the hospital and those who were hospitalized for more than 10 days were excluded to eliminate the patients with the most severe infection, and propensity score matching was conducted to reduce the influencing of potential risk factors, which may result that MSSA infections were more severe. Importantly, death is associated with costly economic loss when loss of production and wages are calculated, however, the indirect costs were not considered in this study. MRSA and MSSA infections are treated empirically using vancomycin before the cultures were available. The earlier optimal therapy for MRSA-related infections would improve outcomes and reduce the healthcare cost, however, it is showed a worse outcomes when MSSA-related infections were treated with vancomycin rather than beta-lactam agents [109]. In addition, inpatients with repeated hospitalizations, repeated operations, and repeated infections, which can often cause a prolonged hospital stay with huge costs are not considered in this study. Thus, this findings need to be interpreted with caution.

There is a vast difference in the excess cost among the same comparison groups in the different countries, even within a single country. First, it may be due to the differences between the healthcare systems in the different countries, especially with regard to the medical pricing, insurance system, and reimbursement policy. Second, the different opinions and traditions regarding how to treat infections in different countries are closely associated with the difference in prescribing patterns of antibiotics [110], which further contributes to the geographic differences in ABR [111]. A lot of regional and national surveillance systems have been built to collect representative and accurate ABR data, in order to provide timely information for policy decisions, such as the European Antimicrobial Resistance Surveillance Network (EARS-Net) and Latin American Surveillance Network of Antimicrobial Resistance (ReLAVRA) [17]. WHO launched the Global Antimicrobial Resistance Surveillance System (GLASS) in 2015, which is the first global collaborative and standardized antimicrobial resistance surveillance system [112]. In addition, differences in study design including type of study, study perspective, study method, study population, source of infection or colonization, sample size, and even description of cost likely account for much of the extreme variation in economic outcomes. As is widely known, societal cost for lost productivity for ABR are greater than direct healthcare costs [10], and death may well save healthcare costs but create a severe cost to society and the family in lost wages and production. Some studies developed economic models such as total factor productivity and using a dynamic general equilibrium model to estimate the loss of productivity due to ABR [14, 113]. Eventually, diverse comparison groups, even in the same bacteria, may result in the differences in comparison of the results in different studies. We find that there is a standard definition for “antibiotic resistance” or “multi-drug resistance”, but they might not be followed in the different studies, which consequently prevents the public from having a complete comprehension of the extent of the problem of ABR. Policy makers cannot get the accurate information about the rising threat of MDR to public health as well. The European Centre for Disease Prevention and Control (ECDC) and the Centers for Disease Control and Prevention (CDC) created a standardized international terminology to define organisms that are resistant to a significant number of antibiotics. However, it only includes *S. aureus*, *Enterococcus spp.*, *Enterobacteriaceae* (other than *Salmonella* and *Shigella*), *P. aeruginosa* and *Acinetobacter spp.*, bacteria. Moreover, the same lists of antibiotic categories proposed for antibiotic susceptibility testing in different hospitals, regions, or countries need to be carefully considered [114].

The ESKAPE organisms, as a serious global problem, have attached a lot of attention. Recently published research focus on gram-positive bacteria, namely, *S. aureus* and *Enterococcus*. However, ABR, especially MDR gram-negative bacteria are becoming increasingly prevalent and constitute a serious threat to global public health because they are difficult to treat and are associated with a substantial economic burden [6, 115]. CRAB, CRPA, and other carbapenem resistant *Enterobacteriaceae* (CRKP and carbapenem resistant *E. coli* (CREC)), are classified as priority 1 (critical) on the WHO priority pathogens list for research and development of new antibiotics against ABR [16]. Further studies identifying the effect of resistance on economic outcomes are critical in prioritizing future therapy for these types of bacteria and in optimizing medical resource to control carbapenem resistance.

Our study was subject to certain limitations. First, since we only included articles in the English and Chinese languages, and published literatures, potential language bias and publication bias cannot be neglected. Second, due to different types of values (mean or median) of costs or charges as the primary outcome, no meta-analyses were performed. Third, the majority of studies have been conducted retrospectively; in contrast to a prospective study, there may exist missing data and selection bias due to the retrospective nature [116]. In addition, most of studies were conducted in a single hospital setting, and only took direct cost into consideration regardless of indirect cost, thus, further studies in prospective design, from multiple hospital settings, and on societal cost for ABR are needed. Last, it is limited to ESKAPE organisms chosen because they are among the most important organisms responsible for ABR, MDR, extensively drug resistance or pan-drug resistance.

## Conclusions

ABR in ESKAPE organisms is not always, but usually, associated with significantly higher economic burden. These results without significant differences may lack statistical power to detect a significant association. Study design which controls for severity of illness and same empirical antibiotic therapy in the two groups would be expected to bias the study towards a similar, even negative result.

There is a vast difference in the excess cost among the same comparison groups in the different countries, even within a single country, which may be due to the different healthcare systems and different opinions and traditions on antibiotic treatments in different countries. Differences in study design and inconsistent standardized definition for ABR and MDR can contribute to diverging results as well.

The review highlights key areas where further researches are needed. Further studies using prospective design, from multiple hospital settings, at a regional and national level are needed. Exploring the loss of production and wages due to ABR or MDR is important for evaluating overall economic burden of ABR. In addition, we should pay more attention to the economic impact of MDR gram-negative bacteria, namely, CRAB, CRPA, CRKP, and CREC.

## Additional files

Additional file 1: Search terms and search strategies. (DOCX 16 kb)

Additional file 2: Study Quality Assessment. **Table S9**. Quality assessment checklist for nonrandomized studies. **Table S10**. Study quality of the included study. (DOCX 120 kb)

Additional file 3: **Table S1**. Studies characteristics associated with resistant and multi-drug resistant Staphylococcus aureus. **Table S2**. Studies characteristics associated with resistant and multi-drug resistant Enterococcus. **Table S3**. Studies characteristics associated with resistant and multi-drug resistant E.coli and Klebsiella spp./ K. pneumoniae. **Table S4**. Studies characteristics associated with resistant and multi-drug resistant E. coli. **Table S5**. Studies characteristics associated with resistant and multi-drug resistant K. pneumoniae. **Table S6**. Studies characteristics associated with resistant and multi-drug resistant P. aeruginosa. **Table S7.** Studies characteristics associated with resistant and multi-drug resistant A. baumannii. **Table S8.** Studies describing hospital costs among patients with resistant or multi-drug resistant ESKAPE organisms according to different organisms and types of infection. (DOCX 143 kb)

## Data Availability

All data analysed during this study are provided in the attached file.

## Abbreviations

ABR: Antibiotic resistance
AMR: Antimicrobial resistance
BSI: Bloodstream infection
CDC: Centers for Disease Control and Prevention
CRAB: Carbapenem resistant *A. baumannii*
CREC: Carbapenem resistant *E. coli*
CRKP: Carbapenem resistant *K. pneumoniae*
CRPA: Carbapenem resistant *P. aeruginosa*
CSAB: Carbapenem susceptible *A. baumannii*
CSPA: Carbapenem susceptible *P. aeruginosa*
EARS-Net: Carbapenem susceptible *A. baumannii*
ECDC: European Centre for Disease Prevention and Control
EEA: European Economic Area
ESBL: Extended spectrum β-lactamases
EU: European Union
GDP: Gross domestic product
GLASS: Global Antimicrobial Resistance Surveillance System
IAI: Intra-abdominal infection
ICU: Intensive care unit
MDR: Multi-drug resistance
MRSA: Methicillin resistant *S. aureus*
MSSA: Methicillin susceptible *S. aureus*
NOS: Newcastle-Ottawa quality assessment Scale
ReLAVRA: Latin American Surveillance Network of Antimicrobial Resistance
US: United States
UTI: Urinary tract infection
VRE: Vancomycin resistant *Enterococcus*
VSE: Vancomycin susceptible *Enterococcus*
WHO: World Health Organization
