# Supplementary material for: Economic burden of antibiotic resistance in ESKAPE organisms: a systematic review
Source: Antimicrob Resist Infect Control. 2019 Aug 13;8:137. doi: 10.1186/s13756-019-0590-7 (PMC6692939; doi:10.1186/s13756-019-0590-7)
Supplement: Supplementary file 1 — Search terms and search strategies. (DOCX 16 kb) [file 13756_2019_590_MOESM1_ESM.docx]

Search terms and search strategies

1. PubMed [5063]

(antimicrobial* [Title/Abstract] OR antibiotic [Title/Abstract] OR microbial [Title/Abstract] OR bacterial*[Title/Abstract] OR multi-drug [Title/Abstract] OR multidrug [Title/Abstract] OR multiple-drug[Title/Abstract] OR multiple drug [Title/Abstract] OR gram-positive[Title/Abstract] OR gram-negative[Title/Abstract] OR enterococcus[Title/Abstract] OR escherichia[Title/Abstract] OR klebsiella[Title/Abstract] OR enterobacter[Title/Abstract] OR pseudomonas[Title/Abstract] OR acinetobacter[Title/Abstract] OR staphylococcus[Title/Abstract]) **AND**

(resistan*[Title/Abstract] OR MDR[Title/Abstract] OR susceptib*[Title/Abstract] OR nonsusceptib*[Title/Abstract]) **AND**

(economic*[Title] OR cost*[Title] OR length of stay[Title] OR hospital stay*[Title] OR mortality[Title] OR clinical[Title] OR resource utilization[Title] OR burden[Title] OR outcome[Title]) **AND**

(inpatients [Mesh terms] OR outpatients [Mesh terms] OR inpatient*[Title/Abstract] OR outpatient*[Title/Abstract] OR hospital[Title/Abstract] OR hospitals[Title/Abstract] OR hospitalization[Title/Abstract] OR hospitalized[Title/Abstract])

2. Web of Science [1250]

(antimicrobial* OR antibiotic OR microbial OR bacterial* OR multi-drug OR multidrug OR multiple-drug OR multiple drug OR gram-positive OR gram-negative OR enterococcus OR escherichia OR klebsiella OR enterobacter OR pseudomonas OR acinetobacter OR staphylococcus) [Topic] **AND**

(resistan* OR MDR OR susceptib* OR nonsusceptib*) [Topic] **AND**

(inpatients OR outpatients OR patients OR patient OR hospital OR hospitals OR hospitalization OR hospitalized) [Topic] **AND**

(economic* OR cost* OR length of stay OR hospital stay* OR mortality OR clinical OR outcome OR burden OR resource utilization) [Title]

3. Embase [7010]

(‘antimicrobial*’:ab,ti OR ‘antibiotic’:ab,ti OR ‘microbial’:ab,ti OR ‘bacterial’:ab,ti OR ‘multi-drug’:ab,ti OR ‘multidrug’:ab,ti OR ‘multiple-drug’:ab,ti OR ‘multiple drug’:ab,ti OR ‘gram-positive’:ab,ti OR ‘gram-negative’:ab,ti OR ‘enterococcus’:ab,ti OR ‘escherichia’:ab,ti OR ‘klebsiella’:ab,ti OR ‘enterobacter’:ab,ti OR ‘pseudomonas’:ab,ti OR ‘acinetobacter’:ab,ti OR ‘staphylococcus’:ab,ti) **AND**

(‘resistan*’:ab,ti OR ‘MDR’:ab,ti OR ‘susceptib*’:ab,ti OR ‘nonsusceptib*’:ab,ti) **AND**

(‘economic*’:ti OR ‘cost*’:ti OR ‘length of stay’:ti OR ‘hospital stay*’:ti OR ‘mortality’:ti OR ‘clinical’:ti OR ‘outcome’:ti OR ‘burden’:ti OR ‘resource utilization’:ti) **AND**

(‘hospital patient’/exp OR ‘inpatients’:ab,ti OR ‘outpatients’:ab,ti OR ‘hospital’:ab,ti OR ‘hospitals’:ab,ti OR ‘hospitalization’:ab,ti OR ‘hospitalized’:ab,ti)

4. CNKI [242]

SU=(’抗生素’+’抗菌药物+’病原菌’+’致病菌’+’抗菌素’+’金黄色葡萄球菌’+’金葡菌’+’肠球菌’+’大肠埃希*’+’大肠杆菌’+’肠杆菌’+’肺炎克雷*’+’铜绿假单胞菌’+’鲍曼不动杆菌’+’鲍氏不动杆菌’+’革兰阴性菌’+’革兰氏阴性*’+’革兰阳性菌’+’革兰氏阳性*’) AND SU=(’耐药’+’敏感’+’多重耐药’+’多药耐药’) AND SU=(‘住院费用’+’医疗费用’+’经济负担’+’疾病负担’+’住院天数’+’住院时间’+’住院日’+’死亡率’+’病死率’) AND SU=(‘患者’+’病人’)

5. Wanfang data [105]

主题:(抗生素+抗菌药物+病原菌+致病菌+抗菌素+金黄色葡萄球菌+金葡菌+肠球菌+”大肠埃希*”+肠杆菌+”肺炎克雷*”+铜绿假单胞菌+鲍曼不动杆菌+鲍氏不动杆菌+革兰阴性菌+革兰氏阴性+革兰阳性菌+革兰氏阳性)*主题:(耐药+敏感+多重耐药+多药耐药)*题名或关键词:(住院费用+医疗费用+经济负担+疾病负担+住院天数+住院时间+住院日+死亡率+病死率)*主题:(患者+病人)

6. CQVIP [23]

(K=(抗生素 OR 抗菌药物 OR 病原菌 OR 致病菌 OR 抗菌素 OR 金黄色葡萄球菌 OR 金葡菌 OR 肠球菌 OR 大肠埃希 OR 肠杆菌 OR 肺炎克雷 OR 铜绿假单胞菌 OR 鲍曼不动杆菌 OR 鲍氏不动杆菌 OR 革兰阴性菌 OR 革兰氏阴性 OR 革兰阳性菌 OR 革兰氏阳性)) AND (K=(耐药 OR 敏感 OR 多重耐药 OR 多药耐药)) AND (K=(住院费用 OR 医疗费用 OR 经济负担 OR 疾病负担 OR 住院天数 OR 住院时间 OR 住院日 OR 死亡率 OR 病死率)) AND (K=(患者 OR病人))
