# Supplementary material for: Economic burden of antibiotic resistance in ESKAPE organisms: a systematic review
Source: Antimicrob Resist Infect Control. 2019 Aug 13;8:137. doi: 10.1186/s13756-019-0590-7 (PMC6692939; doi:10.1186/s13756-019-0590-7)
Supplement: Supplementary file 2 — Study Quality Assessment. Table S9. Quality assessment checklist for nonrandomized studies. Table S10. Study quality of the included study. (DOCX 120 kb) [file 13756_2019_590_MOESM2_ESM.docx]

**Additional file 2: Study Quality Assessment**

Table S9. Quality assessment checklist for nonrandomized studies.

| **Domain** | **Checklist Criteria** | **Additional Information** |
| --- | --- | --- |
| Selection | **1) Is the case definition adequate?**  a) yes, with independent validation *****  b) yes, eg record linkage or based on self-reports  c) no description |  |
|  | **2) Representativeness of the cases**  a) consecutive or obviously representative series of cases *  b) potential for selection biases or not stated | Assumption: Given the nature of the review, studies received a star if they discuss representation (and reasons for their study being representative) or if they are multicentre/regional/national. |
|  | **3) Selection of Controls**  a) community controls *****  b) hospital controls  c) no description | Assumption: It was assumed that if the study was in a hospital setting in which cases were hospital patients, hospital controls were accepted. |
|  | **4) Definition of Controls**  a) no history of disease (endpoint) *****  b) no description of source | Assumption: History of disease/infection was used in this criteria even in studies looking at mortality or other burden outcomes |
| Comparability | **1) Comparability of cases and controls on the basis of the design or analysis**  a) study controls for age/sex/comorbidities *  b) study controls for any additional factor ***** | 2 * maximum allotted for this criteria.  Assumption: For studies in which hospital associated cases and LoS were being analysed, two stars were only given if time dependency was controlled for |
| Exposure | **1) Ascertainment of exposure**  a) secure record (eg surgical records) *****  b) structured interview where blind to case/control status *****  c) interview not blinded to case/control status  d) written self-report or medical record only  e) no description | Assumption: Studies which utilised lab techniques were used to ascertain exposure received one star. |
|  | **2) Same method of ascertainment for cases and controls**  a) yes *****  b) no |  |
|  | **3) Non-Response rate**  a) same rate for both groups *****  b) non respondents described  c) rate different and no designation | Assumption: No description of data cleaning or linkage and loss to missing data for retrospective studies was panelised by not awarding a star |

Table S10. Study quality of the included study.

| **Study** | **Selection 1)**  ***** | **Selection 2)**  ***** | **Selection 3)**  ***** | **Selection 4)**  ***** | **Comparability 1)**  ****** | **Exposure 1)**  ***** | **Exposure 2)**  ***** | **Exposure 3)**  ***** | **Total (MAX. 9)** |
| --- | --- | --- | --- | --- | --- | --- | --- | --- | --- |
| [1] | 1 | 1 | 1 | 1 | 1 | 1 | 1 | 1 | 8 |
| [2] | 1 | 1 | 1 | 1 | 0 | 1 | 1 | 0 | 6 |
| [3] | 1 | 1 | 1 | 1 | 1 | 1 | 0 | 0 | 6 |
| [4] | 1 | 1 | 1 | 1 | 0 | 1 | 1 | 0 | 6 |
| [5] | 1 | 1 | 1 | 1 | 1 | 1 | 1 | 0 | 7 |
| [6] | 1 | 1 | 1 | 1 | 1 | 1 | 1 | 1 | 8 |
| [7] | 1 | 1 | 1 | 1 | 1 | 1 | 0 | 1 | 7 |
| [8] | 1 | 1 | 1 | 1 | 0 | 1 | 1 | 1 | 7 |
| [9] | 1 | 1 | 1 | 1 | 0 | 1 | 1 | 1 | 7 |
| [10] | 1 | 1 | 1 | 1 | 1 | 1 | 1 | 0 | 7 |
| [11] | 1 | 1 | 1 | 1 | 1 | 1 | 1 | 0 | 7 |
| [12] | 1 | 1 | 1 | 1 | 1 | 1 | 0 | 0 | 6 |
| [13] | 1 | 1 | 1 | 1 | 0 | 1 | 1 | 0 | 6 |
| [14] | 1 | 1 | 1 | 1 | 1 | 1 | 1 | 1 | 8 |
| [15] | 1 | 1 | 1 | 1 | 1 | 1 | 1 | 0 | 7 |
| [16] | 1 | 1 | 1 | 1 | 1 | 1 | 1 | 1 | 8 |
| [17] | 1 | 1 | 1 | 1 | 0 | 1 | 1 | 0 | 6 |
| [18] | 1 | 1 | 1 | 1 | 1 | 1 | 1 | 1 | 8 |
| [19] | 1 | 1 | 1 | 1 | 0 | 1 | 1 | 1 | 7 |
| [20] | 1 | 1 | 1 | 1 | 0 | 1 | 1 | 1 | 7 |
| [21] | 1 | 1 | 1 | 1 | 1 | 1 | 0 | 0 | 6 |
| [22] | 1 | 1 | 1 | 1 | 1 | 1 | 1 | 1 | 8 |
| [23] | 1 | 1 | 1 | 1 | 1 | 1 | 1 | 0 | 7 |
| [24] | 1 | 1 | 1 | 1 | 1 | 1 | 1 | 1 | 8 |
| [25] | 1 | 1 | 1 | 1 | 1 | 1 | 1 | 0 | 7 |
| [26] | 1 | 1 | 1 | 1 | 0 | 1 | 1 | 1 | 7 |
| [27] | 1 | 1 | 1 | 1 | 1 | 1 | 1 | 0 | 7 |
| [28] | 1 | 1 | 1 | 1 | 1 | 1 | 1 | 1 | 8 |
| [29] | 1 | 1 | 1 | 1 | 1 | 1 | 1 | 1 | 8 |
| [30] | 1 | 1 | 1 | 1 | 1 | 1 | 1 | 1 | 8 |
| [31] | 1 | 1 | 1 | 1 | 0 | 1 | 1 | 0 | 6 |
| [32] | 1 | 1 | 1 | 1 | 0 | 1 | 1 | 1 | 7 |
| [33] | 1 | 1 | 1 | 1 | 1 | 1 | 1 | 0 | 7 |
| [34] | 1 | 1 | 1 | 1 | 0 | 1 | 1 | 1 | 7 |
| [35] | 1 | 1 | 1 | 1 | 1 | 1 | 1 | 1 | 8 |
| [36] | 1 | 1 | 1 | 1 | 1 | 1 | 1 | 1 | 8 |
| [37] | 1 | 1 | 1 | 1 | 1 | 1 | 1 | 1 | 8 |
| [38] | 1 | 1 | 1 | 1 | 1 | 1 | 1 | 1 | 8 |
| [39] | 1 | 1 | 1 | 1 | 1 | 1 | 0 | 0 | 6 |
| [40] | 1 | 1 | 1 | 1 | 0 | 1 | 1 | 0 | 6 |
| [41] | 1 | 1 | 1 | 1 | 1 | 1 | 1 | 0 | 7 |
| [42] | 1 | 1 | 1 | 1 | 1 | 1 | 0 | 1 | 7 |
| [43] | 1 | 1 | 1 | 1 | 1 | 1 | 1 | 0 | 7 |
| [44] | 1 | 1 | 1 | 1 | 1 | 1 | 1 | 1 | 8 |
| [45] | 1 | 1 | 1 | 1 | 1 | 1 | 1 | 0 | 7 |
| [46] | 1 | 1 | 1 | 1 | 1 | 1 | 1 | 1 | 8 |
| [47] | 1 | 1 | 1 | 1 | 1 | 1 | 0 | 0 | 6 |
| [48] | 1 | 1 | 1 | 1 | 1 | 1 | 1 | 0 | 7 |
| [49] | 1 | 1 | 1 | 1 | 1 | 1 | 0 | 0 | 6 |
| [50] | 1 | 1 | 1 | 1 | 0 | 1 | 1 | 0 | 6 |
| [51] | 1 | 1 | 1 | 1 | 0 | 1 | 1 | 1 | 7 |
| [52] | 1 | 1 | 1 | 1 | 1 | 1 | 1 | 1 | 8 |
| [53] | 1 | 1 | 1 | 1 | 0 | 1 | 1 | 1 | 7 |
| [54] | 1 | 1 | 1 | 1 | 1 | 1 | 0 | 1 | 7 |
| [55] | 1 | 1 | 1 | 1 | 0 | 1 | 0 | 1 | 6 |
| [56] | 1 | 1 | 1 | 1 | 0 | 1 | 1 | 0 | 6 |
| [57] | 1 | 1 | 1 | 1 | 1 | 1 | 1 | 0 | 7 |
| [58] | 1 | 1 | 1 | 1 | 1 | 1 | 1 | 0 | 7 |
| [59] | 1 | 1 | 1 | 1 | 1 | 1 | 1 | 1 | 8 |
| [60] | 1 | 1 | 1 | 1 | 1 | 1 | 1 | 0 | 7 |
| [61] | 1 | 1 | 1 | 1 | 1 | 1 | 1 | 0 | 7 |
| [62] | 1 | 1 | 1 | 1 | 1 | 1 | 1 | 1 | 8 |
| [63] | 1 | 1 | 1 | 1 | 1 | 1 | 1 | 1 | 8 |
| [64] | 1 | 1 | 1 | 1 | 1 | 1 | 1 | 0 | 7 |
| [65] | 1 | 1 | 1 | 1 | 1 | 1 | 1 | 0 | 7 |
| [66] | 1 | 1 | 1 | 1 | 1 | 1 | 1 | 1 | 8 |
| [67] | 1 | 1 | 1 | 1 | 1 | 1 | 1 | 1 | 8 |
| [68] | 1 | 1 | 1 | 1 | 1 | 1 | 1 | 0 | 7 |
| [69] | 1 | 1 | 1 | 1 | 1 | 1 | 1 | 0 | 7 |
| [70] | 1 | 1 | 1 | 1 | 1 | 1 | 1 | 0 | 7 |
| [71] | 1 | 1 | 1 | 1 | 1 | 1 | 1 | 0 | 7 |
| [72] | 1 | 1 | 1 | 1 | 1 | 1 | 0 | 0 | 6 |
| [73] | 1 | 1 | 1 | 1 | 1 | 1 | 1 | 1 | 8 |
| [74] | 1 | 1 | 1 | 1 | 1 | 1 | 1 | 0 | 7 |
| [75] | 1 | 1 | 1 | 1 | 1 | 1 | 1 | 1 | 8 |
| [76] | 1 | 1 | 1 | 1 | 1 | 1 | 1 | 1 | 8 |
| [77] | 1 | 1 | 1 | 1 | 1 | 1 | 1 | 1 | 8 |
| [78] | 1 | 1 | 1 | 1 | 1 | 1 | 1 | 0 | 7 |
| [79] | 1 | 1 | 1 | 1 | 1 | 1 | 1 | 0 | 7 |
| [80] | 1 | 1 | 1 | 1 | 1 | 1 | 1 | 1 | 8 |
| [81] | 1 | 1 | 1 | 1 | 1 | 1 | 0 | 1 | 7 |
| [82] | 1 | 1 | 1 | 1 | 0 | 1 | 0 | 0 | 5 |
| [83] | 1 | 1 | 1 | 1 | 1 | 1 | 1 | 0 | 7 |

**References**

1. Chen W, Li S, Li H, Zhang S, Liu B, Zhang X*, et al***.** Comparison in prognosis of hospital-acquired pneumonia due to methicillin-resistant and methicillin-sensitive Staphylococcus aureus: analysis of propensity score matching. Chinese Journal of Infection Control**.** 2016; 15:299-303.

2. Li X, Chen Y, Gao W, Ouyang W, Wei J, Wen Z**.** Epidemiology and outcomes of complicated skin and soft tissue infections among inpatients in southern China from 2008 to 2013. PLoS One**.** 2016; 11:e149960.

3. Fu J, Chen B, Wang X, Ye Q, Lu Z, Su C*, et al***.** Case-control study of influence of methicillin-resistant Staphylococcus aureus infection on economic losses and length of hospital stay. Chinese Journal of Nosocomiology**.** 2014; 24:2363-5.

4. Xu B, Yuan H, Yang P**.** Evaluation of economic burden induced by multidrug-resistant bacteria related infections in a tertiary general hospital. Chinese Journal of Experimental and Clinical Infectious Diseases**.** 2017; 11:455-9.

5. Lee YJ, Chen JZ, Lin HC, Liu HY, Lin SY, Lin HH*, et al***.** Impact of active screening for methicillin-resistant Staphylococcus aureus (MRSA) and decolonization on MRSA infections, mortality and medical cost: a quasi-experimental study in surgical intensive care unit. Critical Care (London, England)**.** 2015; 19:143.

6. Park SY, Son JS, Oh IH, Choi JM, Lee MS**.** Clinical impact of methicillin-resistant Staphylococcus aureus bacteremia based on propensity scores. Infection**.** 2011; 39:141-7.

7. Kim CJ, Kim HB, Oh MD, Kim Y, Kim A, Oh SH*, et al***.** The burden of nosocomial Staphylococcus aureus bloodstream infection in South Korea: a prospective hospital-based nationwide study. BMC Infectious Diseases**.** 2014; 14:590.

8. Thampi N, Showler A, Burry L, Bai AD, Steinberg M, Ricciuto DR*, et al***.** Multicenter study of health care cost of patients admitted to hospital with Staphylococcus aureus bacteremia: Impact of length of stay and intensity of care. American Journal of Infection Control**.** 2015; 43:739-44.

9. Rubio-Terres C, Garau J, Grau S, Martinez-Martinez L**.** Cost of bacteraemia caused by methicillin-resistant vs. methicillin-susceptible Staphylococcus aureus in Spain: a retrospective cohort study. Clinical Microbiology and Infection**.** 2010; 16:722-8.

10. Resch A, Wilke M, Fink C**.** The cost of resistance: incremental cost of methicillin-resistant Staphylococcus aureus (MRSA) in German hospitals. European Journal of Health Economics**.** 2009; 10:287-97.

11. Ott E, Bange FC, Reichardt C, Graf K, Eckstein M, Schwab F*, et al***.** Costs of nosocomial pneumonia caused by meticillin-resistant Staphylococcus aureus. Journal of Hospital Infection**.** 2010; 76:300-3.

12. Engler-Huesch S, Heister T, Mutters NT, Wolff J, Kaier K**.** In-hospital costs of community-acquired colonization with multidrug-resistant organisms at a German teaching hospital. BMC Health Services Research**.** 2018; 18.

13. de Kraker MEA, Davey PG, Grundmann H**.** Mortality and hospital stay associated with resistant Staphylococcus aureus and Escherichia coli bacteremia: estimating the burden of antibiotic resistance in Europe. PLoS Medicine**.** 2011; 8.

14. Engemann JJ, Carmeli Y, Cosgrove SE, Fowler VG, Bronstein MZ, Trivette SL*, et al***.** Adverse clinical and economic outcomes attributable to methicillin resistance among patients with Staphylococcus aureus surgical site infection. Clinical Infectious Diseases**.** 2003; 36:592-8.

15. Ben-David D, Novikov I, Mermel LA**.** Are there differences in hospital cost between patients with nosocomial methicillin-resistant Staphylococcus aureus bloodstream infection and those with methicillin-susceptible S. aureus bloodstream infection? Infection Control and Hospital Epidemiology**.** 2009; 30:453-60.

16. Kopp BJ, Nix DE, Armstrong EP**.** Clinical and economic analysis of methicillin-susceptible and -resistant Staphylococcus aureus infections. The Annals of Pharmacotherapy**.** 2004; 38:1377-82.

17. Lodise TP, McKinnon PS**.** Clinical and economic impact of methicillin resistance in patients with Staphylococcus aureus bacteremia. Diagnostic Microbiology and Infectious Disease**.** 2005; 52:113-22.

18. Song X, Perencevich E, Campos J, Short BL, Singh N**.** Clinical and economic impact of methicillin-resistant Staphylococcus aureus colonization or infection on neonates in intensive care units. Infection Control and Hospital Epidemiology**.** 2010; 31:177-82.

19. Shorr AF, Haque N, Taneja C, Zervos M, Lamerato L, Kothari S*, et al***.** Clinical and economic outcomes for patients with health care-associated Staphylococcus aureus pneumonia. Journal of Clinical Microbiology**.** 2010; 48:3258-62.

20. Taneja C, Haque N, Oster G, Shorr AF, Zilber S, Kyan PO*, et al***.** Clinical and economic outcomes in patients with community-acquired Staphylococcus aureus pneumonia. Journal of Hospital Medicine**.** 2010; 5:528-34.

21. Anderson DJ, Kaye KS, Chen LF, Schmader KE, Choi Y, Sloane R*, et al***.** Clinical and financial outcomes due to methicillin resistant Staphylococcus aureus surgical site infection: a multi-center matched outcomes study. PLoS One**.** 2009; 4:e8305.

22. Branch-Elliman W, Lee GM, Golen TH, Gold HS, Baldini LM, Wright SB**.** Health and economic burden of post-partum Staphylococcus aureus breast abscess. PLoS One**.** 2013; 8:e73155.

23. Cosgrove SE, Qi Y, Kaye KS, Harbarth S, Karchmer AW, Carmeli Y**.** The impact of methicillin resistance in Staphylococcus aureus bacteremia on patient outcomes: mortality, length of stay, and hospital charges. Infection Control and Hospital Epidemiology**.** 2005; 26:166-74.

24. Filice GA, Nyman JA, Lexau C, Lees CH, Bockstedt LA, Como-Sabetti K*, et al***.** Excess costs and utilization associated with methicillin resistance for patients with Staphylococcus aureus infection. Infection Control and Hospital Epidemiology**.** 2010; 31:365-73.

25. Klein EY, Jiang W, Mojica N, Tseng KK, McNeill R, Cosgrove SE*, et al***.** National costs associated with methicillin-susceptible and methicillin-resistant Staphylococcus aureus hospitalizations in the United States, 2010-2014. Clinical Infectious Diseases**.** 2019; 68:22-8.

26. Shorr AF, Tabak YP, Gupta V, Johannes RS, Liu LZ, Kollef MH**.** Morbidity and cost burden of methicillin-resistant Staphylococcus aureus in early onset ventilator-associated pneumonia. Critical care (London, England)**.** 2006; 10:R97.

27. Reed SD, Friedman JY, Engemann JJ, Griffiths RI, Anstrom KJ, Kaye KS*, et al***.** Costs and outcomes among hemodialysis-dependent patients with methicillin-resistant or methicillin-susceptible Staphylococcus aureus bacteremia. Infection Control and Hospital Epidemiology**.** 2005; 26:175-83.

28. Itani KM, Merchant S, Lin SJ, Akhras K, Alandete JC, Hatoum HT**.** Outcomes and management costs in patients hospitalized for skin and skin-structure infections. American Journal of Infection Control**.** 2011; 39:42-9.

29. Nelson RE, Samore MH, Jones M, Greene T, Stevens VW, Liu CF*, et al***.** Reducing time-dependent bias in estimates of the attributable cost of health care-associated methicillin-resistant Staphylococcus aureus infections: a comparison of three estimation strategies. Medical Care**.** 2015; 53:827-34.

30. Nelson RE, Jones M, Liu CF, Samore MH, Evans ME, Graves N*, et al***.** The impact of healthcare-associated methicillin-resistant Staphylococcus aureus infections on post-discharge healthcare costs and utilization. Infection Control and Hospital Epidemiology**.** 2015; 36:534-42.

31. McHugh CG, Riley LW**.** Risk factors and costs associated with methicillin-resistant Staphylococcus aureus bloodstream infections. Infection Control and Hospital Epidemiology**.** 2004; 25:425-30.

32. Capitano B, Leshem OA, Nightingale CH, Nicolau DP**.** Cost effect of managing methicillin-resistant Staphylococcus aureus in a long-term care facility. Journal of the American Geriatrics Society**.** 2003; 51:10-6.

33. Jiang HL, Zhou Z, Wang LS, Fang Y, Li YH, Chu CI**.** The risk factors, costs, and survival analysis of invasive VRE infections at a medical center in eastern Taiwan. International Journal of Infectious Diseases**.** 2017; 54:18-24.

34. Kramer TS, Remschmidt C, Werner S, Behnke M, Schwab F, Werner G*, et al***.** The importance of adjusting for enterococcus species when assessing the burden of vancomycin resistance: a cohort study including over 1000 cases of enterococcal bloodstream infections. Antimicrobial Resistance and Infection Control**.** 2018; 7:133.

35. Puchter L, Chaberny IF, Schwab F, Vonberg RP, Bange FC, Ebadi E**.** Economic burden of nosocomial infections caused by vancomycin-resistant enterococci. Antimicrobial Resistance and Infection Control**.** 2018; 7:1.

36. Cheah AL, Spelman T, Liew D, Peel T, Howden BP, Spelman D*, et al***.** Enterococcal bacteraemia: factors influencing mortality, length of stay and costs of hospitalization. Clinical Microbiology and Infection**.** 2013; 19:E181-9.

37. Lloyd-Smith P, Younger J, Lloyd-Smith E, Green H, Leung V, Romney MG**.** Economic analysis of vancomycin-resistant enterococci at a Canadian hospital: assessing attributable cost and length of stay. Journal of Hospital infection**.** 2013; 85:54-9.

38. Jung E, Byun S, Lee H, Moon SY, Lee H**.** Vancomycin-resistant Enterococcus colonization in the intensive care unit: clinical outcomes and attributable costs of hospitalization. American Journal of Infection Control**.** 2014; 42:1062-6.

39. Pelz RK, Lipsett PA, Swoboda SM, Diener-West M, Powe NR, Brower RG*, et al***.** Vancomycin-sensitive and vancomycin-resistant enterococcal infections in the ICU: attributable costs and outcomes. Intensive Care Medicine**.** 2002; 28:692-7.

40. Webb M, Riley LW, Roberts RB**.** Cost of hospitalization for and risk factors associated with vancomycin-resistant Enterococcus faecium infection and colonization. Clinical Infectious Diseases**.** 2001; 33:445-52.

41. Carmeli Y, Eliopoulos G, Mozaffari E, Samore M**.** Health and economic outcomes of vancomycin-resistant enterococci. Archives of Internal Medicine**.** 2002; 162:2223-8.

42. Butler AM, Olsen MA, Merz LR, Guth RM, Woeltje KF, Camins BC*, et al***.** Attributable costs of Enterococcal bloodstream infections in a nonsurgical hospital cohort. Infection Control and Hospital Epidemiology**.** 2010; 31:28-35.

43. Ford CD, Lopansri BK, Haydoura S, Snow G, Dascomb KK, Asch J*, et al***.** Frequency, risk factors, and outcomes of vancomycin-resistant Enterococcus colonization and infection in patients with newly diagnosed acute leukemia: different patterns in patients with acute myelogenous and acute lymphoblastic leukemia. Infection Control and Hospital Epidemiology**.** 2015; 36:47-53.

44. Adams DJ, Eberly MD, Goudie A, Nylund CM**.** Rising vancomycin-resistant Enterococcus infections in hospitalized children in the United States. Hospital Pediatrics**.** 2016; 6:404-11.

45. Gearhart M, Martin J, Rudich S, Thomas M, Wetzel D, Solomkin J*, et al***.** Consequences of vancomycin-resistant Enterococcus in liver transplant recipients: a matched control study. Clinical Transplantation**.** 2005; 19:711-6.

46. Nguyen GC, Leung W, Weizman AV**.** Increased risk of vancomycin-resistant Enterococcus (VRE) infection among patients hospitalized for inflammatory bowel disease in the United States. Inflammatory Bowel Diseases**.** 2011; 17:1338-42.

47. Song XY, Srinivasan A, Plaut D, Perl TM**.** Effect of nosocomial vancomycin-resistant enterococcal bacteremia on mortality, length of stay, and costs. Infection Control and Hospital Epidemiology**.** 2003; 24:251-6.

48. Hu B, Ye H, Xu Y, Ni Y, Hu Y, Yu Y*, et al***.** Clinical and economic outcomes associated with community-acquired intra-abdominal infections caused by extended spectrum beta-lactamase (ESBL) producing bacteria in China. Current Medical Research and Opinion**.** 2010; 26:1443-9.

49. Meng X, Liu S, Duan J, Huang X, Zhou P, Xiong X*, et al***.** Risk factors and medical costs for healthcare-associated carbapenem-resistant Escherichia coli infection among hospitalized patients in a Chinese teaching hospital. BMC Infectious Diseases**.** 2017; 17.

50. Huang W, Qiao F, Zhang Y, Huang J, Deng Y, Li J*, et al***.** In-hospital medical costs of infections caused by carbapenem-resistant Klebsiella pneumoniae. Clinical Infectious Diseases**.** 2018; 672:S225-30.

51. Yang Y, Ku C, Lin J, Shang S, Chiu C, Yeh K*, et al***.** Impact of extended-spectrum beta-lactamase-producing Escherichia coli and Klebsiella pneumoniae on the outcome of community-onset bacteremic urinary tract infections. Journal of Microbiology Immunology and Infection**.** 2010; 43:194-9.

52. Apisarnthanarak A, Kiratisin P, Mundy LM**.** Predictors of mortality from community-onset bloodstream infections due to extended-spectrum beta-lactamase-producing Escherichia coli and Klebsiella pneumoniae. Infection Control and Hospital Epidemiology**.** 2008; 29:671-4.

53. Apisarnthanarak A, Kiratisin P, Saifon P, Kitphati R, Dejsirilert S, Mundy LM**.** Predictors of mortality among patients with community-onset infection due to extended-spectrum beta-lactamase producing Escherichia coli in Thailand. Infection Control and Hospital Epidemiology**.** 2008; 29:80-2.

54. Apisarnthanarak A, Kiratisin P, Saifon P, Kitphati R, Dejsirilert S, Mundy LM**.** Clinical and molecular epidemiology of community-onset, extended-spectrum beta-lactamase-producing Escherichia coli infections in Thailand: a case-case-control study. American Journal of Infection Control**.** 2007; 35:606-12.

55. Apisarnthanarak A, Kiratisin P, Saifon P, Kitphati R, Dejsirilert S, Mundy LM**.** Risk factors for and outcomes of healthcare-associated infection due to extended-spectrum beta-lactamase-producing Escherichia coli or Klebsiella pneumoniae in Thailand. Infection Control and Hospital Epidemiology**.** 2007; 28:873-6.

56. Cornejo-Juarez P, Suarez-Cuenca JA, Volkow-Fernandez P, Silva-Sanchez J, Barrios-Camacho H, Najera-Leon E*, et al***.** Fecal ESBL Escherichia coli carriage as a risk factor for bacteremia in patients with hematological malignancies. Supportive Care in Cancer**.** 2016; 24:253-9.

57. Leistner R, Bloch A, Sakellariou C, Gastmeier P, Schwab F**.** Costs and length of stay associated with extended-spectrum β-lactamase production in cases of Escherichia coli bloodstream infection. Journal of global antimicrobial resistance. Journal of Global Antimicrobial Resistance**.**; 3:107-9.

58. Esteve-Palau E, Solande G, Sanchez F, Sorli L, Montero M, Gueerri R*, et al***.** Clinical and economic impact of urinary tract infections caused by ESBL-producing Escherichia coli requiring hospitalization: a matched cohort study. Journal of Infection**.** 2015; 71:667-74.

59. Tumbarello M, Spanu T, Di Bidino R, Marchetti M, Ruggeri M, Trecarichi EM*, et al***.** Costs of bloodstream infections caused by Escherichia coli and influence of extended-spectrum-beta-lactamase production and inadequate initial antibiotic therapy. Antimicrobial Agents and Chemotherapy**.** 2010; 54:4085-91.

60. Maslikowska JA, Walker SAN, Elligsen M, Mittmann N, Palmay L, Daneman N*, et al***.** Impact of infection with extended-spectrum beta-lactamase-producing Escherichia coli or Klebsiella species on outcome and hospitalization costs. Journal of Hospital Infection**.** 2016; 92:33-41.

61. Lautenbach E, Patel JB, Bilker WB, Edelstein PH, Fishman NO**.** Extended-spectrum beta-lactamase-producing Escherichia coli and Klebsiella pneumoniae: risk factors for infection and impact of resistance on outcomes. Clinical Infectious Diseases**.** 2001; 32:1162-71.

62. Lee SY, Kotapati S, Kuti JL, Nightingale CH, Nicolau DP**.** Impact of extended-spectrum beta-lactamase-producing Escherichia coli and Klebsiella species on clinical outcomes and hospital costs: a matched cohort study. Infection Control and Hospital Epidemiology**.** 2006; 27:1226-32.

63. MacVane SH, Tuttle LO, Nicolau DP**.** Impact of extended-spectrum beta-lactamase-producing organisms on clinical and economic outcomes in patients with urinary tract infection. Journal of Hospital Medicine**.** 2014; 9:232-8.

64. Thaden JT, Li Y, Ruffin F, Maskarinec SA, Hill-Rorie JM, Wanda LC*, et al***.** Increased costs associated with bloodstream infections caused by multidrug-resistant gram-negative bacteria are due primarily to patients with hospital-acquired infections. Antimicrobial Agents and Chemotherapy**.** 2017; 61.

65. Alam MF, Cohen D, Butler C, Dunstan F, Roberts Z, Hillier S*, et al***.** The additional costs of antibiotics and re-consultations for antibiotic-resistant Escherichia coli urinary tract infections managed in general practice. International Journal of Antimicrobial Agents**.** 2009; 33:255-7.

66. Chen Z, Xu Z, Wu H, Chen L, Gao S, Chen Y**.** The impact of carbapenem-resistant Pseudomonas aeruginosa on clinical and economic outcomes in a Chinese tertiary care hospital: a propensity score-matched analysis. American Journal of Infection Control**.** 2018.

67. Morales E, Cots F, Sala M, Comas M, Belvis F, Riu M*, et al***.** Hospital costs of nosocomial multi-drug resistant Pseudomonas aeruginosa acquisition. BMC Health Services Research**.** 2012; 12.

68. Lautenbach E, Synnestvedt M, Weiner MG, Bilker WB, Vo L, Schein J*, et al***.** Imipenem resistance in Pseudomonas aeruginosa: emergence, epidemiology, and impact on clinical and economic outcomes. Infection Control and Hospital Epidemiology**.** 2010; 31:47-53.

69. Lautenbach E, Weiner MG, Nachamkin I, Bilker WB, Sheridan A, Fishman NO**.** Imipenem resistance among pseudomonas aeruginosa isolates: risk factors for infection and impact of resistance on clinical and economic outcomes. Infection Control and Hospital Epidemiology**.** 2006; 27:893-900.

70. Gasink LB, Fishman NO, Nachamkin I, Bilker WB, Lautenbach E**.** Risk factors for and impact of infection or colonization with aztreonam-resistant Pseudomonas aeruginosa. Infection Control and Hospital Epidemiology**.** 2007; 28:1175-80.

71. Gasink LB, Fishman NO, Weiner MG, Nachamkin I, Bilker WB, Lautenbach E**.** Fluoroquinolone-resistant Pseudomonas aeruginosa: assessment of risk factors and clinical impact. American Journal of Medicine**.** 2006; 119.

72. Eagye KJ, Kuti JL, Nicolau DP**.** Risk factors and outcomes associated with isolation of meropenem high-level-resistant Pseudomonas aeruginosa. Infection Control and Hospital Epidemiology**.** 2009; 30:746-52.

73. Cui N, Cao B, Liu Y, Liang L, Gu L, Song S**.** The impact of imipenem-resistant Acinetobacter baumannii infection on clinical outcomes and medical care costs. Chinese Journal of Infectious Diseases**.** 2012; 30:209-14.

74. Guo Y, Guo W, Qiu P, He Q, Pan C, Wu C*, et al***.** Study of attributive hospitalized cost and length of stay for hospital acquired infection due to multidrug resistance and none-resistance Acinetobacter baumannii. Chinese Journal of Health Statistics**.** 2017; 34:378-81.

75. Wu X, Ding L, Wu X**.** Direct economic loss due to healthcare-associated infection with multidrug resistant Acinetobacter baumannii. Chinese Journal of Infection Control**.** 2018; 17:735-8.

76. Zhen X, Chen Y, Hu X, Dong P, Gu S, Sheng YY*, et al***.** The difference in medical costs between carbapenem-resistant Acinetobacter baumannii and non-resistant groups: a case study from a hospital in Zhejiang province, China. European Journal of Clinical Microbiology & Infectious Diseases**.** 2017; 36:1989-94.

77. Lee NY, Lee HC, Ko NY, Chang CM, Shih HI, Wu CJ*, et al***.** Clinical and economic impact of multidrug resistance in nosocomial Acinetobacter baumannii bacteremia. Infection Control and Hospital Epidemiology**.** 2007; 28:713-9.

78. Thatrimontrichai A, Techato C, Dissaneevate S, Janjindamai W, Maneenil G, Kritsaneepaiboon S*, et al***.** Risk factors and outcomes of carbapenem-resistant Acinetobacter baumannii ventilator-associated pneumonia in the neonate: a case-case-control study. Journal of Infection and Chemotherapy**.** 2016; 22:444-9.

79. Lemos EV, de la Hoz FP, Alvis N, Einarson TR, Quevedo E, Castaneda C*, et al***.** Impact of carbapenem resistance on clinical and economic outcomes among patients with Acinetobacter baumannii infection in Colombia. Clinical Microbiology and Infection**.** 2014; 20:174-80.

80. Lee H, Lee H**.** Clinical and economic evaluation of multidrug-resistant Acinetobacter baumannii colonization in the intensive care unit. Infection & Chemotherapy**.** 2016; 48:174-80.

81. Young LS, Sabel AL, Price CS**.** Epidemiologic, clinical, and economic evaluation of an outbreak of clonal multidrug-resistant Acinetobacter baumannii infection in a surgical intensive care unit. Infection Control and Hospital Epidemiology**.** 2007; 28:1247-54.

82. Wilson SJ, Knipe CJ, Zieger MJ, Gabehart KM, Goodman JE, Volk HM*, et al***.** Direct costs of multidrug-resistant Acinetobacter baumannii in the burn unit of a public teaching hospital. American Journal of Infection Control**.** 2004; 32:342-4.

83. Lautenbach E, Synnestvedt M, Weiner MG, Bilker WB, Vo L, Schein J*, et al***.** Epidemiology and impact of imipenem resistance in Acinetobacter baumannii. Infection Control and Hospital Epidemiology**.** 2009; 30:1186-92.
