# Supplementary material for: Economic burden of antibiotic resistance in ESKAPE organisms: a systematic review
Source: Antimicrob Resist Infect Control. 2019 Aug 13;8:137. doi: 10.1186/s13756-019-0590-7 (PMC6692939; doi:10.1186/s13756-019-0590-7)
Supplement: Supplementary file 3 — Table S1. Studies characteristics associated with resistant and multi-drug resistant Staphylococcus aureus. Table S2. Studies characteristics associated with resistant and multi-drug resistant Enterococcus. Table S3. Studies characteristics associated with resistant and multi-drug resistant E.coli and Klebsiella spp./ K. pneumoniae. Table S4. Studies characteristics associated with resistant and multi-drug resistant E. coli. Table S5. Studies characteristics associated with resistant and multi-drug resistant K. pneumoniae. Table S6. Studies characteristics associated with resistant and multi-drug resistant P. aeruginosa. Table S7. Studies characteristics associated with resistant and multi-drug resistant A. baumannii. Table S8. Studies describing hospital costs among patients with resistant or multi-drug resistant ESKAPE organisms according to different organisms and types of infection. (DOCX 143 kb) [file 13756_2019_590_MOESM3_ESM.docx]

**Additional file 3**

**Table S1. Studies characteristics associated with resistant and multi-drug resistant *Staphylococcus aureus*.**

| **Author** | **Year** | **Type of study** | **Country** | **Study setting** | **Study period** | **Method** | **Study population** | **Infection/**  **colonization** | **Hospital/**  **community-acquired** | **Infection type** | **Hospital ward** | **Bacteria** | **Comparison** | | **Sample size** | | **Description of cost** |
| --- | --- | --- | --- | --- | --- | --- | --- | --- | --- | --- | --- | --- | --- | --- | --- | --- | --- |
|  |  |  |  |  |  |  |  |  |  |  |  |  | **Case** | **Control** | **Case** | **Control** |  |
| Chen et al. [1] | 2016 | Retrospective case-control | China | Single | 2013-2014 | Significant test | Adult patients ≥18 years | Infection | Hospital-acquired | Pneumonia | Not mentioned | S. aureus | MRSA | MSSA | 75 | 78 | Total hospital cost |
|  |  |  |  |  |  | Propensity score matching/ significant test |  |  |  |  |  | S. aureus | MRSA | MSSA | 46 | 46 | Total hospital cost |
| Shorr et al. [2] | 2010 | Retrospective | US | Single | 2005-2008 | Significant test | Not mentioned | Infection | Hospital-acquired | Pneumonia | Not mentioned | S. aureus | MRSA | MSSA | 87 | 55 | Total hospital charge |
| Taneja et al. [3] | 2010 | Retrospective | US | Single | 2005-2008 | Significant test | Not mentioned | Infection | Community-acquired | Pneumonia | Not mentioned | S. aureus | MRSA | MSSA | 55 | 73 | Total hospital charge |
| Shorr et al. [4] | 2006 | Retrospective cohort | US | Multiple | 2002-2003 | Significant test | Not mentioned | Infection | Not mentioned | Ventilator associated pneumonia | Not mentioned | S. aureus | MRSA | MSSA | 59 | 95 | Total hospital cost |
| Itani et al. [5] | 2011 | Retrospective | US | Multiple | 2002-2006 | Multivariate linear regression | Adult inpatients ≥18 years | Infection | Not mentioned | Skin and soft tissue infection | Not mentioned | S. aureus | MRSA | MSSA | 1114 | 4042 | Total hospital charge |
| Li et al. [6] | 2016 | Retrospective | China | Multiple | 2008-2013 | Significant test | Not mentioned | Infection | Not mentioned | Complicated skin and soft tissue infection | Not mentioned | S. aureus | MRSA | MSSA | 14 | 61 | Total hospital cost |
| Park et al. [7] | 2011 | Retrospective case-control | South Korea | Single | 2003-2008 | Propensity scores matching/ significant test | Not mentioned | Infection | Hospital-acquired | Bacteremia | Not mentioned | S. aureus | MRSA | MSSA | 53 | 53 | Total hospital cost |
| Klein et al. [8] | 2019 | Retrospective | US | Multiple | 2010-2014 | Propensity scores matching/ significant test | Not mentioned | Infection | Not mentioned | Septicemia | Not mentioned | S. aureus | MRSA | MSSA | 54255 | 57065 | Total hospital cost |
|  |  |  |  |  |  |  |  |  |  | Pneumonia |  | S. aureus | MRSA | MSSA | 48780 | 25020 | Total hospital cost |
|  |  |  |  |  |  |  |  |  |  | Not mentioned |  | S. aureus | MRSA | MSSA | 255105 | 175845 | Total hospital cost |
| Branch-Elliman et al. [9] | 2013 | Retrospective cohort | US | Single | 2003-2010 | Matching/ significant test | Pregnant women with breast abscess | Not mentioned | Not mentioned | Breast abscess | Not mentioned | S. aureus | MRSA | MSSA | 30 | 24 | Total hospital cost |
| Kopp et al. [10] | 2004 | Retrospective case-control | US | Single | 1999-2000 | Matching/ significant test | Not mentioned | Infection | Not mentioned | Not mentioned | Not mentioned | S. aureus | MRSA | MSSA | 36 | 36 | Total hospital cost |
|  |  |  |  |  |  |  |  |  |  |  |  | S. aureus | MRSA | MSSA | 36 | 36 | Total hospital charge |
| de Kraker et al. [11] | 2011 | Prospective cohort | Europe | Multiple | 2007 | Significant test | Not mentioned | Not mentioned | Not mentioned | Bacteremia | Not mentioned | S. aureus | MRSA | MSSA | 27711 | 80723 | Excess total hospital cost |
| Ott et al. [12] | 2010 | Retrospective case-control | Germany | Single | 2005-2007 | Matching/ significant test | Not mentioned | Not mentioned | Hospital-acquired | Pneumonia | Not mentioned | S. aureus | MRSA | MSSA | 41 | 41 | Total hospital cost |
| Ben-David et al. [13] | 2009 | Retrospective cohort | US | Single | 2000-2003 | Significant test | Not mentioned | Infection | Hospital-acquired | Bloodstream infection | ICU | S. aureus | MRSA | MSSA | 42 | 34 | Total hospital cost |
|  |  |  |  |  |  |  |  |  |  |  | general ward | S. aureus | MRSA | MSSA | 53 | 53 | Total hospital cost |
|  |  |  |  |  |  |  |  |  |  |  | ICU | S. aureus | MRSA | MSSA | 42 | 34 | Hospital cost after culture |
|  |  |  |  |  |  |  |  |  |  |  | General ward | S. aureus | MRSA | MSSA | 53 | 53 | Hospital cost after culture |
| McHugh et al. [14] | 2004 | Retrospective case-control | US | Single | 1997-1999 | Significant test | Not mentioned | Infection | Not mentioned | Bloodstream infection | Not mentioned | S. aureus | MRSA | MSSA | 20 | 40 | Total hospital charge |
| Thampi et al. [15] | 2015 | Retrospective cohort | Canada | Multiple | 2007-2010 | Significant test | Adult inpatients ≥18 years | Not mentioned | Not mentioned | Bacteremia | Not mentioned | S. aureus | MRSA | MSSA | 58 | 377 | Total hospital charge |
| Rubio-Terres et al. [16] | 2010 | Retrospective cohort | Spain | Multiple | 2005 | Significant test | Adult patients ≥18 years | Not mentioned | Not mentioned | Bacteremia | Not mentioned | S. aureus | MRSA | MSSA | 121 | 245 | Total hospital cost |
| Reed et al. [17] | 2005 | Prospective cohort | US | Single | 1996-2001 | Propensity scores matching/ significant test | Hemodialysis-dependent adult patients ≥18 years | Infection | Not mentioned | Bacteremia | Not mentioned | S. aureus | MRSA | MSSA | 54 | 89 | Total hospital cost |
| Engemann et al. [18] | 2003 | Prospective cohort | US | Multiple | 1994-2000 | Significant test | Patients with surgical procedures | Infection | Not mentioned | Surgical site infection | Not mentioned | S. aureus | MRSA | MSSA | 121 | 165 | Total hospital charge |
|  |  |  |  |  |  | Significant test |  |  |  |  |  | S. aureus | MRSA | Without infection | 121 | 193 | Total hospital charge |
|  |  |  |  |  |  | Multivariate linear regression |  |  |  |  |  | S. aureus | MRSA | MSSA | 121 | 165 | Total hospital charge |
| Anderson et al. [19] | 2009 | Prospective | US | Multiple | 1998-2003 | Significant test | Not mentioned | Infection | Not mentioned | Surgical site infection | Not mentioned | S. aureus | MRSA | Without infection | 150 | 231 | Total hospital charge |
|  |  |  |  |  |  |  |  |  |  |  |  | S. aureus | MRSA | MSSA | 150 | 128 | Total hospital charge |
| Song et al. [20] | 2010 | Retrospective cohort | US | Single | 2004-2008 | Multivariate linear regression | Newborns and infants | Colonizaiton | Not mentioned | Not mentioned | Neonatal ICU | S. aureus | MRSA | MSSA | 128 | 2089 | Excess total hospital charge |
|  |  |  |  |  |  |  |  | Infection |  |  |  | S. aureus | MRSA | MSSA | 63 | 2089 | Excess total hospital charge |
| Filice et al. [21] | 2010 | Retrospective | US | Single | 2004-2006 | Semilogarithmic orginary least-squares model | Adult inpatients and outpatients ≥18 years | Infection | Not mentioned | Not mentioned | Not mentioned | S. aureus | MRSA | MSSA | 335 | 390 | Total hospital cost |
|  |  |  |  |  |  |  |  |  |  |  |  | S. aureus | MRSA | MSSA | 335 | 390 | Antibiotic cost |
| Nelson et al. [22] | 2015 | Retrospective cohort | US | Single | 2007-2010 | Propensity scores matching/ multivariable regression model | Not mentioned | Infection | Hospital-acquired | Not mentioned | Not mentioned | S. aureus | MRSA | MSSA | 3592 | 3592 | Total hospital cost |
| Lee et al. [23] | 2015 | Retrospective | Taiwan, China | Single | 2007-2010 | Significant test | Not mentioned | Infection | Not mentioned | Not mentioned | Surgical ICU | S. aureus | MRSA | MSSA | 25 | 25 | Total hospital cost |
|  |  |  |  |  |  |  |  |  |  |  |  | S. aureus | MRSA | MSSA | 25 | 25 | ICU cost |
| Resch et al. [24] | 2009 | Retrospective | Germany | Multiple | 2004 | Matching 1/ significant test | Not mentioned | Not mentioned | Not mentioned | Not mentioned | Not mentioned | S. aureus | MRSA | MSSA | 1026 | 1026 | Total hospital cost |
|  |  |  |  |  |  | Matching 2/ significant test |  |  |  |  |  | S. aureus | MRSA | MSSA | 549 | 549 | Total hospital cost |
| Nelson et al. [25] | 2015 | Retrospective cohort | US | Single | 2007-2010 | Conventional analysis: generalized linear model | Not mentioned | Infection | Hospital-acquired | Not mentioned | Not mentioned | S. aureus | MRSA | MSSA | 3982 | 382812 | Excess total hospital cost |
|  |  |  |  |  |  | Post-hospital-acquired infection: generalized linear model |  |  |  |  |  | S. aureus | MRSA | MSSA | 92 | 121428 | Excess total hospital cost |
|  |  |  |  |  |  | Propensity scores matching/ generalized linear model |  |  |  |  |  | S. aureus | MRSA | MSSA | 2872 | 10120 | Excess total hospital cost |
| Xu et al. [26] | 2017 | Retrospective | China | Single | 2015 | Significant test | Not mentioned | Infection | Not mentioned | Not mentioned | Not mentioned | S. aureus | MRSA | MSSA | 41 | 237 | Total hospital cost |
|  |  |  |  |  |  |  |  |  |  |  |  | S. aureus | MRSA | MSSA | 41 | 237 | Antibiotic cost |
| Capitano et al. [27] | 2003 | Retrospective cohort | US | Single | 1996-2000 | Significant test | Not mentioned | Infection | Not mentioned | Not mentioned | Not mentioned | S. aureus | MRSA | MSSA | 41 | 49 | Total infection cost |
| Cosgrove et al. [28] | 2005 | Prospective cohort | US | Single | 1997-2000 | Significant test | Not mentioned | Not mentioned | Not mentioned | Bloodstream infection | Not mentioned | S. aureus | MRSA | MSSA | 96 | 252 | Hospital charge after culture |
|  |  |  |  |  |  |  |  |  |  |  |  | S. aureus | MRSA | MSSA | 96 | 252 | Hospital cost after culture |
|  |  |  |  |  |  |  |  |  |  |  |  | S. aureus | MRSA | MSSA | 96 | 252 | Hospital charge before culture |
| Lodise et al. [29] | 2005 | Retrospective cohort | US | Single | 1999-2001 | Significant test | Not mentioned | Not mentioned | Not mentioned | Bacteremia | Not mentioned | S. aureus | MRSA | MSSA | 170 | 183 | Hospital cost after culture |
|  |  |  |  |  |  | Analysis of covariance |  |  |  |  |  | S. aureus | MRSA | MSSA | 170 | 183 | Hospital cost after culture |
| Kim et al. [30] | 2014 | Prospective case-control | South Korea | Multiple | 2011 | Matching/ significant test | Adult inpatients ≥18 years | Infection | Hospital-acquired | Bloodstream infection | Not mentioned | S. aureus | MRSA | Without infection | 133 | 133 | Total hospital cost |
|  |  |  |  |  |  |  |  |  |  |  |  | S. aureus | MRSA | Without infection | 133 | 133 | Total hospital charge |
| Fu et al. [31] | 2014 | Retrospective case-control | China | Single | 2012-2013 | Matching/ significant test | Not mentioned | Infection | Not mentioned | Not mentioned | Not mentioned | S. aureus | MRSA | Without infection | 456 | 706 | Total hospital cost |
| Engler Husch et al. [32] | 2018 | Retrospective case-cohort | Germany | Single | 2011-2014 | Matching/ generalized linear model | Adult inpatients ≥18 years | Colonization | Community-acquired | Not mentioned | Not mentioned | S. aureus | MRSA | Without colonizaiton | 453 | 7917 | Excess total hospital cost |
| MRSA: methicillin resistant *S. aureus*; MSSA: methicillin susceptible *S. aureus*; ICU: intensive care unit.  **Table S2. Studies characteristics associated with resistant and multi-drug resistant *Enterococcus*.** | | | | | | | | | | | | | | | | | |
| **Author** | **Year** | **Type of study** | **Country** | **Study setting** | **Study period** | **Method** | **Study population** | **Infection/colonization** | **Hospital/community-acquired** | **Infection type** | **Hospital ward** | **Bacteria** | **Comparison** | | **Sample size** | | **Description of cost** |
|  |  |  |  |  |  |  |  |  |  |  |  |  | **Case** | **Control** | **Case** | **Control** |  |
| Butler et al. [33] | 2010 | Retrospective cohort | US | Single | 2002-2003 | Multivariate generalized least squares model/ propensity score matching | Non-surgical patients | Infection | Not mentioned | Bloodstream infection | Not mentioned | Enterococcus | VRE | VSE | 94 | 182 | Total hospital cost |
|  |  |  |  |  |  |  |  |  |  |  |  | Enterococcus | VRE | Without infection | 94 | 20150 | Total hospital cost |
| Ford et al. [34] | 2015 | Retrospective case-control | US | Single | 2006-2012 | Matching/ significant test | Patients with acute leukemia | Colonization or infection | Not mentioned | Bloodstream infection | Not mentioned | Enterococcus | VRE | VSE | 15 | 45 | Total hospital cost |
| Kramer et al. [35] | 2018 | Retrospective cohort | Germany | Multiple | 2008-2015 | Significant test | Not mentioned | Infection | Not mentioned | Bloodstream infection | Not mentioned | E. faecium | VRE | VSE | 103 | 493 | Total hospital cost |
| Cheah et al. [36] | 2013 | Retrospective cohort | Australia | Multiple | 2002-2010 | Matching/ significant test | Adult patients ≥18 years and not pregnant | Not mentioned | Not mentioned | Bacteraemia | Not mentioned | Enterococcus | VRE | VSE | 116 | 116 | Total hospital cost |
| Lloyd-Smith et al. [37] | 2013 | Retrospective case-control | Canada | Single | 2008-2009 | Significant test | Adult patients ≥18 years and not pregnant patients | Colonization or infection | Not mentioned | Not mentioned | Not mentioned | Enterococcus | VRE | VSE | 217 | 1075 | Total hospital cost |
|  |  |  |  |  |  | Generalized linear model |  |  |  |  |  | Enterococcus | VRE | VSE | 217 | 1075 | Excess total hospital cost |
| Adams et al. [38] | 2016 | Retrospective case-control | US | Multiple | 1991/2000/2003/2006/2009/2012 | Propensity score matching/ significant test | Children and adolescents ≤18 years | Infection | Not mentioned | Not mentioned | Not mentioned | Enterococcus | VRE | VSE | 3356 | 39505704 | Total hospital cost |
| Gearhart et al. [39] | 2005 | Retrospective case-control | US | Multiple | 1995-2002 | Matching/ significant test | Liver transplant inpatients | Infection | Not mentioned | Not mentioned | Not mentioned | Enterococcus | VRE | VSE | 19 | 38 | Total hospital cost |
| Webb et al. [40] | 2001 | Retrospective case-control | US | Single | 1995-1996 | Significant test | Not mentioned | Colonization and infection | Not mentioned | Not mentioned | Not mentioned | E. faecium | VRE | VSE | 262 | 157 | Total hospital cost |
| Carmeli et al. [41] | 2002 | Retrospective cohort | US | Single | 1993-1997 | Propensity score matching/ multivariate linear regression | Not mentioned | Not mentioned | Not mentioned | Not mentioned | Not mentioned | Enterococcus | VRE | VSE | 233 | 647 | Total hospital charge |
| Nguyen et al. [42] | 2011 | Retrospective | US | Multiple | 1998-2004 | Significant test | Inpatients with inflammatory bowel disease | Infection | Not mentioned | Not mentioned | Not mentioned | Enterococcus | VRE | VSE | 123 | 116719 | Total hospital charge |
| Jung et al. [43] | 2014 | Retrospective cohort | South Korea | Single | 2008-2010 | Propensity score matching/ multivariate linear regression | Not mentioned | Colonization | Not mentioned | Not mentioned | ICU | Enterococcus | VRE | VSE | 199 | 199 | Total hospital cost |
|  |  |  |  |  |  |  |  |  |  |  |  | Enterococcus | VRE | VSE | 199 | 199 | ICU cost |
| Puchter et al. [44] | 2018 | Retrospective case-control | Germany | Single | 2005-2008 | Matching/ significant test | Not mentioned | Infection | Hospital-acquired | Not mentioned | Not mentioned | Enterococcus | VRE | VSE | 42 | 42 | Total hospital cost |
|  |  |  |  |  |  |  |  |  |  |  |  | Enterococcus | VRE | VSE | 42 | 42 | Hospital cost before culture |
|  |  |  |  |  |  |  |  |  |  |  |  | Enterococcus | VRE | VSE | 42 | 42 | Hospital cost after culture |
| Jiang et al. [45] | 2017 | Retrospective case-control | Taiwan, China | Single | 2007-2012 | Matching/ significant test | Adult inpatients ≥18 years | Infection | Not mentioned | Not mentioned | Not mentioned | Enterococcus | VRE | VSE | 48 | 142 | Antibiotic cost |
| Pelz et al. [46] | 2002 | Prospective cohort | US | Single | 1996 | Multivariate linear regression | Not mentioned | Infection | Not mentioned | Not mentioned | ICU | Enterococcus | VRE | Without infection | 12 | 89 | ICU cost |
| Song et al. [47] | 2003 | Retrospective cohort | US | Single | 1993-2000 | Matching/ significant test | Not mentioned | Not mentioned | Hospital-acquired | Bacteremia | Not mentioned | Enterococcus | VRE | Without infection | 277 | 277 | Total hospital charge |
| Engler Husch et al. [32] | 2018 | Retrospective case-cohort | Germany | Single | 2011-2014 | Matching/ general linear model | Adult inpatients ≥18 years | Colonization | Community-acquired | Not mentioned | Not mentioned | Enterococcus | VRE | Without colonizaiton | 1004 | 7917 | Excess total hospital cost |
| VRE: vancomycin resistant *Enterococcus*; VSE: vancomycin susceptible *Enterococcus*; ICU: intensive care unit.    **Table S3. Studies characteristics associated with resistant and multi-drug resistant *E.coli and Klebsiella spp./ K. pneumoniae.*** | | | | | | | | | | | | | | | | | |
| **Author** | **Year** | **Type of study** | **Country** | **Study setting** | **Study period** | **Method** | **Study population** | **Infection/colonization** | **Hospital/community-acquired** | **Infection type** | **Hospital ward** | **Bacteria** | **Comparison** | | **Sample size** | | **Description of cost** |
|  |  |  |  |  |  |  |  |  |  |  |  |  | **Case** | **Control** | **Case** | **Control** |  |
| Maslikowska et al. [48] | 2016 | Retrospective case-control | Canada | Single | 2010-2013 | Matching/ significant test | Adult inpatients ≥18 years | Infection | Not mentioned | Not mentioned | Not mentioned | E. coli and Klebsiella spp. | ESBL positive | ESBL negative | 75 | 75 | Total hospital cost (direct and indirect) |
|  |  |  |  |  |  |  |  |  |  |  |  | E. coli and Klebsiella spp. | ESBL positive | ESBL negative | 75 | 75 | Total hospital cost |
| Hu et al. [49] | 2010 | Retrospective | China | Multiple | 2006-2007 | Generalized linear model | Not mentioned | Infection | Community-acquired | Intra-abdominal infection | Not mentioned | E. coli and Klebsiella spp. | ESBL positive | ESBL negative | 32 | 53 | Total hospital cost |
| MacVane et al. [50] | 2014 | Retrospective cohort | US | Single | 2011-2012 | Matching/ significant test | Adult inpatients ≥18 years | Infection | Not mentioned | Urinary tract infection | Not mentioned | E. coli and Klebsiella spp. | ESBL positive | ESBL negative | 55 | 55 | Total hospital cost |
|  |  |  |  |  |  |  |  |  |  |  |  | E. coli and Klebsiella spp. | ESBL positive | ESBL negative | 55 | 55 | Antibiotic cost |
| Yang et al. [51] | 2010 | Retrospective | Taiwan, China | Single | 2006-2008 | Significant test | Adult inpatients ≥18 years | Infection | Community-acquired | Bacteremic urinary tract infection | Not mentioned | E. coli and K. pneumoniae | ESBL positive | ESBL negative | 12 | 46 | Antibiotic cost |
| Apisarnthanarak et al. [52] | 2008 | Retrospective cohort | Thailand | Single | 2003-2007 | Significant test | Adult inpatients ≥18 years | Infection | Community-acquired | Bloodstream infection | Not mentioned | E. coli and K. pneumoniae | ESBL positive | ESBL negative | 36 | 108 | Hospital cost after culture |
| Lautenbach et al. [53] | 2001 | Retrospective case-control | US | Single | 1997-1998 | Matching/ significant test | Not mentioned | Infection | Not mentioned | Not mentioned | Not mentioned | E. coli and K. pneumoniae | ESBL positive | ESBL negative | 33 | 66 | Hospital charge after culture |
| Lee et al. [54] | 2006 | Retrospective cohort | US | Single | 2001-2004 | Matching/ significant test | Not mentioned | Infection | Not mentioned | Not mentioned | Not mentioned | E. coli and Klebsiella spp. | ESBL positive | ESBL negative | 21 | 21 | Total infection cost |
| Apisarnthanarak et al. [55] | 2007 | Retrospective case-control | Thailand | Single | 2003-2004 | Significant test | Adult inpatients ≥18 years | Infection | Hospital-acquired | Not mentioned | Not mentioned | E. coli and K. pneumoniae | ESBL positive | ESBL negative | 74 | 74 | Total hospital cost |
|  |  |  |  |  |  |  |  |  |  |  |  | E. coli and K. pneumoniae | ESBL positive | Without infection | 74 | 74 | Total hospital cost |
| ESBL: extended-spectrum β-lactamases.  **Table S4. Studies characteristics associated with resistant and multi-drug resistant *E. coli.*** | | | | | | | | | | | | | | | | | |
| **Author** | **Year** | **Type of study** | **Country** | **Study setting** | **Study period** | **Method** | **Study population** | **Infection/colonization** | **Hospital/community-acquired** | **Infection type** | **Hospital ward** | **Bacteria** | **Comparison** | | **Sample size** | | **Description of cost** |
|  |  |  |  |  |  |  |  |  |  |  |  |  | **Case** | **Control** | **Case** | **Control** |  |
| de Kraker et al. [11] | 2011 | Prospective cohort | Europe | Multiple | 2007 | Significant test | Not mentioned | Not mentioned | Not mentioned | Bacteremia | Not mentioned | E. coli | Third generation cephalosporin resistant E. coli | Third generation cephalosporin susceptible E. coli | 15183 | 148293 | Excess total hospital cost |
| Tumbarello et al. [56] | 2010 | Retrospective cohort | Italy | Single | 2006 | Multivariate probablistic sensitivity analysis/ significant test | Adult inpatients ≥18 years | Infection | Not mentioned | Bloodstream infection | Not mentioned | E. coli | ESBL positive | ESBL negative | 37 | 97 | Total hospital cost |
| Thaden et al. [57] | 2017 | Prospective cohort | US | Single | 2009-2015 | Generalized linear model | Adult inpatients ≥18 years | Infection | Hospital-acquired | Bloodstream infection | Not mentioned | E. coli | MDR | Non-MDR | 165 | 165 | Total hospital cost |
| Apisarnthanarak et al. [58] | 2008 | Retrospective case-control | Thailand | Single | 2003-2004 | Matching/ significant test | Adult inpatients ≥18 years | Infection | Community-acquired | Not mentioned | Not mentioned | E. coli | ESBL positive | ESBL negative | 46 | 138 | Hospital cost after culture |
| Apisarnthanarak et al. [59] | 2007 | reteospective case control | Thailand | Single | 2003-2004 | Matching/ significant test | Adult inpatients ≥18 years | Infection | Community-acquired | Not mentioned | Not mentioned | E. coli | ESBL positive | Without infection | 46 | 138 | Total hospital cost |
| Alam et al. [60] | 2009 | Retrospective | UK | Multiple | 2002-2004 | Multivariate linear regression | Not mentioned | Infection | Not mentioned | Urinary tract infection | Not mentioned | E. coli | Resistant to at least one antibiotic | Sensitive to all six antibiotics | 389 | 476 | Total hospital cost |
|  |  |  |  |  |  |  |  |  |  |  |  | E. coli | Resistant to at least one antibiotic | Sensitive to all six antibiotics | 389 | 476 | Antibiotic cost |
| Esteve-Palau et al. [61] | 2015 | Retrospective cohort | Spain | Single | 2010-2013 | Matching/ significant test | Adult patients >18 years | Not mentioned | Not mentioned | Urinary tract infection | Not mentioned | E. coli | ESBL positive | ESBL negative | 60 | 60 | Total hospital cost |
|  |  |  |  |  |  |  |  |  |  |  |  | E. coli | ESBL positive | ESBL negative | 60 | 60 | Antibiotic cost |
| Cornejo-Juarez et al. [62] | 2016 | Prospective cohort | Mexico | Single | 2012-2014 | Significant test | Adult patients ≥18 years with hematologica malignancies and severe neutropenia | Colonization | Not mentioned | Not mentioned | Not mentioned | E. coli | ESBL positive | ESBL negative | 63 | 63 | Total hospital cost |
|  |  |  |  |  |  |  |  |  |  |  |  | E. coli | ESBL positive | ESBL negative | 63 | 63 | Antibiotic cost |
| Xu et al. [26] | 2017 | Retrospective | China | Single | 2015 | Significant test | Not mentioned | Infection | Not mentioned | Not mentioned | Not mentioned | E. coli | MDR | Non-MDR | 969 | 1940 | Total hospital cost |
|  |  |  |  |  |  |  |  |  |  |  |  | E. coli | MDR | Non-MDR | 969 | 1940 | Antibiotic cost |
| Meng et al. [63] | 2017 | Retrospective case-control | China | Single | 2012-2015 | Significant test | Not mentioned | Infection | Hospital-acquired | Not mentioned | Not mentioned | E. coli | CREC | CSEC | 49 | 96 | Total hospital cost |
|  |  |  |  |  |  |  |  |  |  |  |  | E. coli | CREC | Without infection | 49 | 96 | Total hospital cost |
| Leistner et al. [64] | 2014 | Retrospective case-control | Germany | Single | 2008-2010 | Matching/ significant test | Not mentioned | Infection | Not mentioned | Bloodstream infection | Not mentioned | E. coli | ESBL positive | ESBL negative | 92 | 92 | Total hospital cost |
| ESBL: extended-spectrum β-lactamases; MDR: multi-drug resistance; CREC: carbapenem resistace *E. coli*; CSEC: carbapenem susceptible *E. coli*.  **Table S5. Studies characteristics associated with resistant and multi-drug resistant *K. pneumoniae.*** | | | | | | | | | | | | | | | | | |
| **Author** | **Year** | **Type of study** | **Country** | **Study setting** | **Study period** | **Method** | **Study population** | **Infection/colonization** | **Hospital/community-acquired** | **Infection type** | **Hospital ward** | **Bacteria** | **Comparison** | | **Sample size** | | **Description of cost** |
|  |  |  |  |  |  |  |  |  |  |  |  |  | **Case** | **Control** | **Case** | **Control** |  |
| Thaden et al. [57] | 2017 | Prospective cohort | US | Single | 2009-2015 | Generalized linear model | Adult inpatients ≥18 years | Infection | Hospital-acquired | Bloodstream infection | Not mentioned | K. pneumoniae | MDR | Non-MDR | 39 | 127 | Total hospital cost |
| Xu et al. [26] | 2017 | Retrospective | China | Single | 2015 | Significant test | Not mentioned | Infection | Not mentioned | Not mentioned | Not mentioned | K. pneumoniae | MDR | Non-MDR | 186 | 529 | Total hospital cost |
|  |  |  |  |  |  |  |  |  |  |  |  | K. pneumoniae | MDR | non-MDR | 186 | 529 | Antibiotic cost |
| Huang et al. [65] | 2018 | Retrospective cohort | China | Single | 2017 | Propensity score matching/ significant test | Not mentioned | Infection | Not mentioned | Not mentioned | Not mentioned | K. pneumoniae | CRKP | CSKP | 237 | 237 | Total hospital cost |
|  |  |  |  |  |  |  |  |  |  |  |  | K. pneumoniae | CRKP | CSKP | 237 | 237 | Hospital cost after culture |
|  |  |  |  |  |  |  |  |  |  |  |  | K. pneumoniae | CRKP | CSKP | 237 | 237 | Antibitoic cost |
| MDR: multi-drug resistance; CRKP: carbapenem resistace *K. pneumoniae*; CSEC: carbapenem susceptible *K. pneumoniae*.  **Table S6. Studies characteristics associated with resistant and multi-drug resistant *P. aeruginosa.*** | | | | | | | | | | | | | | | | | |
| **Author** | **Year** | **Type of study** | **Country** | **Study setting** | **Study period** | **Method** | **Study population** | **Infection/colonization** | **Hospital/community-acquired** | **Infection type** | **Hospital ward** | **Bacteria** | **Comparison** | | **Sample size** | | **Description of cost** |
|  |  |  |  |  |  |  |  |  |  |  |  |  | **Case** | **Control** | **Case** | **Control** |  |
| Chen et al. [66] | 2018 | Retrospective cohort | China | Single | 2014-2018 | Significant test | Adult inpatients ≥18 years | Infection | Not mentioned | Not mentioned | Not mentioned | P. aeruginosa | CRPA | CSPA | 327 | 472 | Total hospital cost |
|  |  |  |  |  |  | Propensity score matching/ significant test |  |  |  |  |  | P. aeruginosa | CRPA | CSPA | 270 | 270 | Total hospital cost |
| Lautenbach et al. [67] | 2006 | Retrospective cohort | US | Single | 1999-2000 | Multivariate linear regression | Adult inpatients ≥18 years | Infection | Not mentioned | Not mentioned | Not mentioned | P. aeruginosa | IRPA | ISPA | 135 | 719 | Total hospital cost |
| Gasink et al. [68] | 2006 | Retrospective cohort | US | Single | 1991-2000 | Significant test | Not mentioned | Not mentioned | Not mentioned | Not mentioned | Not mentioned | P. aeruginosa | Fluoroquinolones resistant P. aeruginosa | Fluoroquinolones susceptible P. aeruginosa | 320 | 527 | Total hospital charge |
| Morales et al. [69] | 2012 | Retrospective | Spain | Single | 2005-2006 | Generalized linear model | Not mentioned | Not mentioned | Hospital-acquired | Not mentioned | Not mentioned | P. aeruginosa | MDR | Non-resistant | 134 | 149 | Total hospital cost |
|  |  |  |  |  |  |  |  |  |  |  |  | P. aeruginosa | Resistant | Non-resistant | 119 | 149 | Total hospital cost |
| Xu et al. [26] | 2017 | Retrospective | China | Single | 2015 | Significant test | Not mentioned | Infection | Not mentioned | Not mentioned | Not mentioned | P. aeruginosa | MDR | Non-MDR | 13 | 490 | Total hospital cost |
|  |  |  |  |  |  |  |  |  |  |  |  | P. aeruginosa | MDR | Non-MDR | 13 | 490 | Antibiotic cost |
| Lautenbach et al. [70] | 2010 | Retrospective cohort | US | Multiple | 2001-2006 | Significant test | Adult inpatients ≥18 years | Colonization or infection | Not mentioned | Not mentioned | Not mentioned | P. aeruginosa | IRPA | ISPA | 253 | 2289 | Hospital cost after culture |
| Gasink et al. [71] | 2007 | Retrospective case-control | US | Single | 1999-2000 | Significant test | Not mentioned | Colonization and infection | Not mentioned | Not mentioned | Not mentioned | P. aeruginosa | Aztreonam resistant P. aeruginosa | Aztreonam susceptible P . aeruginosa | 183 | 537 | Hospital charge after culture |
| Eagye et al. [72] | 2009 | Retrospective case-control | US | Single | 2006 | Significant test | Not mentioned | Not mentioned | Not mentioned | Not mentioned | Not mentioned | P. aeruginosa | MRPA | MSPA | 58 | 125 | Total cost (direct and indirect) |
|  |  |  |  |  |  |  |  |  |  |  |  | P. aeruginosa | MRPA | Without infection | 58 | 57 | Total cost (direct and indirect) |
| CRPA: carbapenem resitance *P. aeruginosa*; CSPA: carbapenem susceptible *P. aeruginosa*; IRPA: imipenem resitance *P. aeruginosa*; ISPA: imipenem susceptible *P. aeruginosa*; MRPA: meropenem resitance *P. aeruginosa*; MSPA: meropenem susceptible *P. aeruginosa*; MDR: multi-drug resistance.  **Table S7. Studies characteristics associated with resistant and multi-drug resistant *A. baumannii.*** | | | | | | | | | | | | | | | | | |
| **Author** | **Year** | **Type of study** | **Country** | **Study setting** | **Study period** | **Method** | **Study population** | **Infection/colonization** | **Hospital/community-acquired** | **Infection type** | **Hospital ward** | **Bacteria** | **Comparison** | | **Sample size** | | **Description of cost** |
|  |  |  |  |  |  |  |  |  |  |  |  |  | **Case** | **Control** | **Case** | **Control** |  |
| Cui et al. [73] | 2012 | Retrospective cohort | China | Single | 2007-2009 | Matching/ significant test | Not mentioned | Infection | Hospital-acquired | Not mentioned | Not mentioned | A. baumannii | IRAB | ISAB | 138 | 138 | Total hospital cost |
|  |  |  |  |  |  |  |  |  |  |  |  | A. baumannii | IRAB | ISAB | 138 | 138 | Antibiotic cost |
| Zhen et al. [74] | 2017 | Retrospective | China | Single | 2013-2015 | Multivariate linear regression | Not mentioned | Infection or colonization | Not mentioned | Not mentioned | Not mentioned | A. baumannii | CRAB | CSAB | 2126 | 854 | Total hospital cost |
|  |  |  |  |  |  |  |  |  |  |  |  | A. baumannii | CRAB | CSAB | 2126 | 854 | Antibiotic cost |
| Lemos et al. [75] | 2014 | Prospective cohort | Colombia | Multiple | 2006-2010 | Backward selection multivariate model | Adult inpatients ≥18 years | Infection | Not mentioned | Not mentioned | ICU | A. baumannii | CRAB | CSAB | 104 | 61 | Total hospital cost |
|  |  |  |  |  |  | Backward selection multivariate model |  |  |  |  |  | A. baumannii | CRAB | CSAB | 104 | 61 | Antibiotic cost |
|  |  |  |  |  |  | Significant test |  |  |  |  |  | A. baumannii | CRAB | CSAB | 104 | 61 | Total hospital cost |
|  |  |  |  |  |  | Significant test |  |  |  |  |  | A. baumannii | CRAB | CSAB | 104 | 61 | Antibiotic cost |
| Lautenbach et al. [76] | 2009 | Retrospective cohort | US | Multiple | 2001-2006 | Significant test | Adult inpatients ≥18 years | Infection or colonization | Not mentioned | Not mentioned | Not mentioned | A. baumannii | IRAB | ISAB | 89 | 297 | Hospital charge after culture |
| Lee et al. [77] | 2016 | Retrospective cohort | South Korea | Single | 2012-2014 | Propensity score matching/ significant test | Not mentioned | Colonization | Not mentioned | Not mentioned | ICU | A. baumannii | MDR | Non-MDR | 122 | 122 | Total hospital cost |
|  |  |  |  |  |  |  |  |  |  |  |  | A. baumannii | MDR | Non-MDR | 122 | 122 | ICU cost |
| Wu et al. [78] | 2018 | Retrospective cohort | China | Single | 2014-2016 | Matching/ significant test | Not mentioned | Infection | Not mentioned | Not mentioned | Not mentioned | A. baumannii | MDR | Non-MDR | 65 | 65 | Total hospital cost |
| Guo et al. [79] | 2017 | Retrospective cohort | China | Single | 2008-2013 | Propensity score matching/ significant test | Not mentioned | Infection | Hospital-acquired | Not mentioned | Not mentioned | A. baumannii | MDR | Non-MDR | 122 | 366 | Total hospital cost |
| Xu et al. [26] | 2017 | Retrospective | China | Single | 2015 | Significant test | Not mentioned | Infection | Not mentioned | Not mentioned | Not mentioned | A. baumannii | MDR | Non-MDR | 53 | 45 | Total hospital cost |
|  |  |  |  |  |  |  |  |  |  |  |  | A. baumannii | MDR | Non-MDR | 53 | 45 | Antibiotic cost |
| Lee et al. [80] | 2007 | Retrospective cohort | Taiwan, China | Single | 1996-2001 | Matching/ significant test | Adult inpatients ≥18 years | Infection | Hospital-acquired | Bacteremia | Not mentioned | A. baumannii | MDR | Non-MDR | 46 | 46 | Total hospital cost |
|  |  |  |  |  |  |  |  |  |  |  |  | A. baumannii | MDR | Non-MDR | 46 | 46 | Antibiotic cost |
| Thatrimontrichai et al. [81] | 2016 | Retrospective case-control | Thailand | Single | 2009-2014 | Matching/ significant test | Infants | Infection | Not mentioned | Ventilator associated pneumonia | ICU | A. baumannii | CRAB | CSAB | 63 | 13 | Total hospital cost |
|  |  |  |  |  |  |  |  |  |  |  |  | A. baumannii | CRAB | Without infection | 63 | 25 | Total hospital cost |
| Young et al [82] | 2007 | Retrospective case-control | US | Single | 2004-2005 | Matching/ significant test | Adult inpatients ≥18 years | Infection | Not mentioned | Not mentioned | Surgical ICU | A. baumannii | MDR | Without infection | 67 | 67 | Total hospital charge |
|  |  |  |  |  |  | Multivariate linear regression |  |  |  |  |  | A. baumannii | MDR | Without infection | 67 | 67 | Total hospital charge |
| Wilson et al. [83] | 2004 | Retrospective case-control | US | Single | 2000-2001 | Significant test | Adult inpatients ≥18 years | Not mentioned | Not mentioned | Not mentioned | Burn ward | A. baumannii | MDR | Without infection | 34 | 34 | Total hospital cost |

CRAB: carbapenem resitance *A. baumannii*; CSAB: carbapenem susceptible *A. baumannii*; IRAB: imipenem resitance *A. baumannii*; ISAB: imipenem susceptible *A. baumannii*; MDR: multi-drug resistance.

**Table S8 Studies describing hospital costs among patients with resistant or multi-drug resistant ESKAPE organisms according to different organisms and types of infection.**

| **Author** | **Method** | **Infection type** | **Hospital ward** | **Bacteria** | **Comparison** | | **Sample size** | | **Description of cost** | **Median cost in 2015 USD** | | | | | | **Mean cost in 2015 USD** | | | | | |
| --- | --- | --- | --- | --- | --- | --- | --- | --- | --- | --- | --- | --- | --- | --- | --- | --- | --- | --- | --- | --- | --- |
|  |  |  |  |  | **Case** | **Control** | **Case** | **Control** |  | **Median cost** | **Case** | **Control** | **Increase** | **Ratio** | **P-value** | **Mean cost** | **Case** | **Control** | **Increase** | **Ratio** | **P-value** |
| Park et al. [7] | Propensity scores matching/ significant test | Bacteremia | Not mentioned | S. aureus | MRSA | MSSA | 53 | 53 | Total hospital cost |  |  |  |  |  |  | Mean (SD) | 10319(14220) | 14220(9867) |  |  | 0.6200 |
| Klein et al. [8] | Propensity scores matching/ significant test | Septicemia | Not mentioned | S. aureus | MRSA | MSSA | 54255 | 57065 | Total hospital cost |  |  |  |  |  |  | Mean (95% CI) | 34561(32951-36170) | 32951(32379-36038) |  |  | 0.6900 |
| Klein et al. [8] | Propensity scores matching/ significant test | Pneumonia | Not mentioned | S. aureus | MRSA | MSSA | 48780 | 25020 | Total hospital cost |  |  |  |  |  | 0.0450 | Mean (95% CI) | 38600(36848-40766) | 36848(38756-42776) | -2166(-1908-(-2010)) | 0.95 | 0.0450 |
| de Kraker et al. [11] | Significant test | Bacteremia | Not mentioned | S. aureus | MRSA | MSSA | 27711 | 80723 | Excess total hospital cost | |  |  |  |  |  | Mean (95% CI) | 2481(1348-3826) | 1348(1933-3110) |  |  | P>0.05 |
| Ott et al. [12] | Matching/ significant test | Pneumonia | Not mentioned | S. aureus | MRSA | MSSA | 41 | 41 | Total hospital cost | Median (IQR) | 94921(36175-146202) | 60583(24034-74790) | 34339(12141-71412) | 1.57 | 0.0110 |  |  |  |  |  |  |
| Ben-David et al. [13] | Significant test | Bloodstream infection | ICU | S. aureus | MRSA | MSSA | 42 | 34 | Total hospital cost | Median (IQR) | 146716(63094-70878) | 54300(41737-96367) | 92416(2135725490) | 2.70 | <0.001 |  |  |  |  |  |  |
| Ben-David et al. [13] | Significant test | Bloodstream infection | general ward | S. aureus | MRSA | MSSA | 53 | 53 | Total hospital cost | Median (IQR) | 68826(42455-108316) | 45272(23634-65588) | 23554(18821-42728) | 1.52 | 0.0050 |  |  |  |  |  |  |
| Ben-David et al. [13] | Significant test | Bloodstream infection | ICU | S. aureus | MRSA | MSSA | 42 | 34 | Hospital cost after culture | Median (IQR) | 66356(31617-134664) | 22684(13180-54274) | 43671(18437-80389) | 2.93 | <0.001 |  |  |  |  |  |  |
| Ben-David et al. [13] | Significant test | Bloodstream infection | General ward | S. aureus | MRSA | MSSA | 53 | 53 | Hospital cost after culture | Median (IQR) | 30528(17455-55896) | 23392(14293-42786) |  |  | 0.3000 |  |  |  |  |  |  |
| McHugh et al. [14] | Significant test | Bloodstream infection | Not mentioned | S. aureus | MRSA | MSSA | 20 | 40 | Total hospital charge |  |  |  |  |  |  | Mean | 65320 | 13797 | 51523 | 4.73 | 0.0003 |
| Thampi et al. [15] | Significant test | Bacteremia | Not mentioned | S. aureus | MRSA | MSSA | 58 | 377 | Total hospital charge | Median (IQR) | 19743(9490-42061) | 12195(7116-29294) | 7547(2374-12767) | 1.62 | 0.0294 |  |  |  |  |  |  |
| Rubio-Terres et al. [16] | Significant test | Bacteremia | Not mentioned | S. aureus | MRSA | MSSA | 121 | 245 | Total hospital cost |  |  |  |  |  |  | Mean | 16669 | 14850 | 1819 | 1.12 | <0.05 |
| Reed et al. [17] | Propensity scores matching/ significant test | Bacteremia | Not mentioned | S. aureus | MRSA | MSSA | 54 | 89 | Total hospital cost | Median (range) | 34763(17499-60252) | 20103(11995-26845) | 14660(5505-33407) | 1.73 | 0.0001 | Mean (SD) | 43715(33886) | 33886(24001) | 18544(9885) | 1.74 | 0.0001 |
| Cosgrove et al. [28] | Significant test | Bloodstream infection | Not mentioned | S. aureus | MRSA | MSSA | 96 | 252 | Hospital charge after culture | Median (IQR) | 36347(19265-69442) | 26426(13754-50272) | 9920(5512-19169) | 1.38 | 0.0080 |  |  |  |  |  |  |
| Cosgrove et al. [28] | Significant test | Bloodstream infection | Not mentioned | S. aureus | MRSA | MSSA | 96 | 252 | Hospital cost after culture | Median (IQR) | 20158(10685-38512) | 14656(7627-27882) | 5502(3058-10630) | 1.38 | 0.0080 |  |  |  |  |  |  |
| Cosgrove et al. [28] | Significant test | Bloodstream infection | Not mentioned | S. aureus | MRSA | MSSA | 96 | 252 | Hospital charge before culture | Median (IQR) | 2868(0-37301) | 1382(0-23414) | 1486(0-13887) | 2.07 | 0.0400 |  |  |  |  |  |  |
| Lodise et al. [29] | Significant test | Bacteremia | Not mentioned | S. aureus | MRSA | MSSA | 170 | 183 | Hospital cost after culture | |  |  |  |  |  | Mean | 30435 | 15000 | 15435 | 2.03 | <0.001 |
| Lodise et al. [29] | Analysis of covariance | Bacteremia | Not mentioned | S. aureus | MRSA | MSSA | 170 | 183 | Hospital cost after culture | |  |  |  |  |  | Mean (95% CI) | 28885(22839-36533) | 22839(12784-19040) | 13265(10055-17493) | 1.85 | 0.0010 |
| Kim et al. [30] | Matching/ significant test | Bloodstream infection | Not mentioned | S. aureus | MRSA | Without infection | 133 | 133 | Total hospital cost | Median (IQR) | 20356(10381-315530) | 8462(5267-18686) | 11895(5115-296844) | 2.41 | <0.05 |  |  |  |  |  |  |
| Kim et al. [30] | Matching/ significant test | Bloodstream infection | Not mentioned | S. aureus | MRSA | Without infection | 133 | 133 | Total hospital charge | Median (IQR) | 4911(2360-8387) | 2486(917-4350) | 2426(1444-4037) | 1.98 | <0.05 |  |  |  |  |  |  |
| Butler et al. [33] | Multivariate generalized least squares model/ propensity score matching | Bloodstream infection | Not mentioned | Enterococcus | VRE | VSE | 94 | 182 | Total hospital cost | Median (IQR) | 48121(18640-107280) | 23880(1443-47862) | 24241(17197-59418) | 2.02 | <0.001 |  |  |  |  |  |  |
| Butler et al. [33] | Multivariate generalized least squares model/ propensity score matching | Bloodstream infection | Not mentioned | Enterococcus | VRE | Without infection | 94 | 20150 | Total hospital cost | Median (IQR) | 48121(18640-107280) | 9362(6417-15423) | 38759(12223-91857) | 5.14 | <0.001 |  |  |  |  |  |  |
| Ford et al. [34] | Matching/ significant test | Bloodstream infection | Not mentioned | Enterococcus | VRE | VSE | 15 | 45 | Total hospital cost | Median | 177503 | 88751 | 88751 | 2.00 | 0.0003 |  |  |  |  |  |  |
| Kramer et al. [35] | Significant test | Bloodstream infection | Not mentioned | E. faecium | VRE | VSE | 103 | 493 | Total hospital cost | Median (IQR) | 89241(53110-174619) | 56967(24993-132854) | 32274(28117-41765) | 1.57 | <0.000 |  |  |  |  |  |  |
| Cheah et al. [36] | Matching/ significant test | Bacteraemia | Not mentioned | Enterococcus | VRE | VSE | 116 | 116 | Total hospital cost | Median (IQR) | 86286(47297-170205) | 43051(27497-99883) | 43235(19800-70322) | 2.00 | 0.0020 |  |  |  |  |  |  |
| Song et al. [47] | Matching/ significant test | Bacteremia | Not mentioned | Enterococcus | VRE | Without infection | 277 | 277 | Total hospital charge | Median | 170917 | 64235 | 106682 | 2.66 | <0.05 |  |  |  |  |  |  |
| Apisarnthanarak et al. [52] | Significant test | Bloodstream infection | Not mentioned | E. coli or K. pneumoniae | ESBL positive | ESBL negative | 36 | 108 | Hospital cost after culture | Median (range) | 703(49-3626) | 245(61-2127) | 458(-11-1499) | 2.87 | <0.001 |  |  |  |  |  |  |
| de Kraker et al. [11] | Significant test | Bacteremia | Not mentioned | E. coli | Third generation cephalosporin resistant E. coli | Third generation cephalosporin susceptible E. coli | 15183 | 148293 | Excess total hospital cost |  |  |  |  |  |  | Mean (95% CI) | 1861(776-3319) | 776(530-1236) | 985(246-2084) | 2.12 | <0.05 |
| Tumbarello et al. [56] | Multivariate probablistic sensitivity analysis/ significant test | Bloodstream infection | Not mentioned | E. coli | ESBL positive | ESBL negative | 37 | 97 | Total hospital cost |  |  |  |  |  |  | Mean (SD) | 13709(16312) | 16312(6683) | 5026(9629) | 1.58 | 0.0300 |
| Thaden et al. [57] | Generalized linear model | Bloodstream infection | Not mentioned | E. coli | MDR | Non-MDR | 165 | 165 | Total hospital cost | Median (IQR) | 9527(5915-19648) | 7503(4301-13447) | 2024(1614-6201) | 1.27 | <0.0001 | Mean (SD) | 18917(29394) | 29394(24416) | 4141(4978) | 1.28 | <0.0001 |
| Leistner et al. [64] | Matching/ significant test | Bloodstream infection | Not mentioned | E. coli | ESBL positive | ESBL negative | 92 | 92 | Total hospital cost | Median (IQR) | 21712(9016-59726) | 23841(8060-67701) |  |  | 0.3590 |  |  |  |  |  |  |
| Thaden et al. [57] | Generalized linear model | Bloodstream infection | Not mentioned | K. pneumoniae | MDR | Non-MDR | 39 | 127 | Total hospital cost | Median (IQR) | 46934(12470-153881) | 11183(5955-29452) | 35751(6515-124429) | 4.20 | 0.0300 | Mean (SD) | 115868(163881) | 163881(48116) | 86991(115765) | 4.01 | 0.0300 |
| Lee et al. [80] | Matching/ significant test | Bacteremia | Not mentioned | A. baumannii | MDR | Non-MDR | 46 | 46 | Total hospital cost |  |  |  |  |  |  | Mean (SD) | 12515(8465) | 8465(5375) | 6003(3090) | 1.92 | 0.0010 |
| Lee et al. [80] | Matching/ significant test | Bacteremia | Not mentioned | A. baumannii | MDR | Non-MDR | 46 | 46 | Antibiotic cost |  |  |  |  |  |  | Mean (SD) | 3021(1822) | 1822(1760) | 866(62) | 1.40 | 0.0140 |
| Chen et al. [1] | Significant test | Pneumonia | Not mentioned | S. aureus | MRSA | MSSA | 75 | 78 | Total hospital cost | Median (IQR) | 23933(14148-34484) | 19905(10916-39283) |  |  | 0.3950 |  |  |  |  |  |  |
| Chen et al. [1] | Propensity score matching/ significant test | Pneumonia | Not mentioned | S. aureus | MRSA | MSSA | 46 | 46 | Total hospital cost | Median (IQR) | 19718(13157-28710) | 19538(10916-3941) |  |  | 0.9350 |  |  |  |  |  |  |
| Shorr et al. [2] | Significant test | Pneumonia | Not mentioned | S. aureus | MRSA | MSSA | 87 | 55 | Total hospital charge | Median | 77123 | 78399 |  |  | >0.05 | Mean (SD) | 108117(104303) | 104303(100566) |  |  | >0.05 |
| Taneja et al. [3] | Significant test | Pneumonia | Not mentioned | S. aureus | MRSA | MSSA | 55 | 73 | Total hospital charge | Median | 79150 | 93164 |  |  | 0.5100 | Mean (SD) | 129393(145555) | 145555(187275) |  |  | 0.5100 |
| Shorr et al. [4] | Significant test | Ventilator associated pneumonia | Not mentioned | S. aureus | MRSA | MSSA | 59 | 95 | Total hospital cost | Median (IQR) | 52492(23643-91577) | 47066(20024-92887) |  |  | 0.3000 |  |  |  |  |  |  |
| Thatrimontrichai et al. [81] | Matching/ significant test | Ventilator associated pneumonia | ICU | A. baumannii | CRAB | CSAB | 63 | 13 | Total hospital cost | Median (range) | 11785(2176-45664) | 9745(5266-24170) |  |  | >0.05 |  |  |  |  |  |  |
| Thatrimontrichai et al. [81] | Matching/ significant test | Ventilator associated pneumonia | ICU | A. baumannii | CRAB | Without infection | 63 | 25 | Total hospital cost | Median (range) | 11785(2176-45664) | 7806(3877-33608) |  |  | >0.05 |  |  |  |  |  |  |
| MacVane et al. [50] | Matching/ significant test | Urinary tract infection | Not mentioned | E. coli or Klebsiella spp. | ESBL positive | ESBL negative | 55 | 55 | Total hospital cost | Median (IQR) | 11085(7065-16325) | 7310(5848-12025) | 3775(1217-4300) | 1.52 | 0.0200 |  |  |  |  |  |  |
| MacVane et al. [50] | Matching/ significant test | Urinary tract infection | Not mentioned | E. coli or Klebsiella spp. | ESBL positive | ESBL negative | 55 | 55 | Antibiotic cost | Median (IQR) | 55(35-82) | 7(6-12) | 48(29-69) | 7.58 | <0.001 |  |  |  |  |  |  |
| Yang et al. [51] | Significant test | Bacteremic urinary tract infection | Not mentioned | E. coli or K. pneumoniae | ESBL positive | ESBL negative | 12 | 46 | Antibiotic cost |  |  |  |  |  |  | Mean (SD) | 677(466) | 466(296) | 399(170) | 2.43 | 0.0140 |
| Alam et al. [60] | Multivariate linear regression | Urinary tract infection | Not mentioned | E. coli | Resistant to at least one antibiotic | Sensitive to all six antibiotics | 389 | 476 | Total hospital cost |  |  |  |  |  |  | Mean (SD) | 33(46) | 46(42) | 8(4) | 1.33 | 0.0060 |
| Alam et al. [60] | Multivariate linear regression | Urinary tract infection | Not mentioned | E. coli | Resistant to at least one antibiotic | Sensitive to all six antibiotics | 389 | 476 | Antibiotic cost |  |  |  |  |  |  | Mean (SD) | 7(14) | 14(3) | 3(11) | 1.72 | <0.001 |
| Esteve-Palau et al. [61] | Matching/ significant test | Urinary tract infection | Not mentioned | E. coli | ESBL positive | ESBL negative | 60 | 60 | Total hospital cost | Median (IQR) | 4877(3537-9645) | 3379(2318-5832) | 1498(1220-3813) | 1.44 | 0.0070 |  |  |  |  |  |  |
| Esteve-Palau et al. [61] | Matching/ significant test | Urinary tract infection | Not mentioned | E. coli | ESBL positive | ESBL negative | 60 | 60 | Antibiotic cost | Median (IQR) | 259(53-605) | 22(8-82) | 238(45-523) | 12.00 | <0.001 |  |  |  |  |  |  |
| Engemann et al. [18] | Significant test | Surgical site infection | Not mentioned | S. aureus | MRSA | MSSA | 121 | 165 | Total hospital charge | Median (IQR) | 127047(55293-187729) | 72615(39992-126279) | 54432(15301-61450) | 1.75 | <0.001 |  |  |  |  |  |  |
| Engemann et al. [18] | Significant test | Surgical site infection | Not mentioned | S. aureus | MRSA | Without infection | 121 | 193 | Total hospital charge | Median (IQR) | 127047(55293-187729) | 40516(21509-57447) | 86531(33784-130282) | 3.14 | <0.001 |  |  |  |  |  |  |
| Engemann et al. [18] | Multivariate linear regression | Surgical site infection | Not mentioned | S. aureus | MRSA | MSSA | 121 | 165 | Total hospital charge | Median (IQR) |  |  |  |  | 0.0300 |  |  |  |  |  |  |
| Anderson et al. [19] | Significant test | Surgical site infection | Not mentioned | S. aureus | MRSA | Without infection | 150 | 231 | Total hospital charge | Median (IQR) | 101841(49115-164750) | 49916(22878-78128) | 51925(26237-86622) | 2.04 | <0.0001 |  |  |  |  |  |  |
| Anderson et al. [19] | Significant test | Surgical site infection | Not mentioned | S. aureus | MRSA | MSSA | 150 | 128 | Total hospital charge | Median (IQR) | 101841(49115-164750) | 71736(28610-111800) | 30106(20505-52950) | 1.42 | 0.0010 |  |  |  |  |  |  |
| Itani et al. [5] | Multivariate linear regression | Skin and soft tissue infection | Not mentioned | S. aureus | MRSA | MSSA | 1114 | 4042 | Total hospital charge | Median | 25741 | 24311 |  |  | 0.4879 | Mean (SD) | 58228(96845) | 96845(139202) |  |  | 0.5921 |
| Li et al. [6] | Significant test | Complicated skin and soft tissue infection | Not mentioned | S. aureus | MRSA | MSSA | 14 | 61 | Total hospital cost | Median | 1977 | 2183 |  |  | 0.3900 | Mean (SD) | 5305(8817) | 8817(3017) |  |  | 0.3900 |
| Branch-Elliman et al. [9] | Matching/ significant test | Breast abscess | Not mentioned | S. aureus | MRSA | MSSA | 30 | 24 | Total hospital cost |  |  |  |  |  |  | Mean (95% CI) |  |  |  |  | 0.4500 |
| Hu et al. [49] | Generalized linear model | Intra-abdominal infection | Not mentioned | E. coli or Klebsiella spp. | ESBL positive | ESBL negative | 32 | 53 | Total hospital cost |  |  |  |  |  |  | Mean | 4119 | 2308 | 1811 | 1.78 | <0.001 |

MRSA: methicillin resistant *S. aureus*; MSSA: methicillin susceptible *S. aureus*; VRE: vancomycin resistant *Enterococcus*; VSE: vancomycin susceptible *Enterococcus*; ESBL: extended-spectrum β-lactamases; CRAB: carbapenem resitance *A. baumannii*; CSAB: carbapenem susceptible *A. baumannii*; MDR: multi-drug resistance; IQR: interquartile range; USD: the United States Dollars; SD: standard deviation; CI: confidence interval.

**References**

1. Chen W, Li S, Li H, Zhang S, Liu B, Zhang X*, et al***.** Comparison in prognosis of hospital-acquired pneumonia due to methicillin-resistant and methicillin-sensitive Staphylococcus aureus: analysis of propensity score matching. Chinese Journal of Infection Control**.** 2016; 15:299-303.

2. Shorr AF, Haque N, Taneja C, Zervos M, Lamerato L, Kothari S*, et al***.** Clinical and economic outcomes for patients with health care-associated Staphylococcus aureus pneumonia. Journal of Clinical Microbiology**.** 2010; 48:3258-62.

3. Taneja C, Haque N, Oster G, Shorr AF, Zilber S, Kyan PO*, et al***.** Clinical and economic outcomes in patients with community-acquired Staphylococcus aureus pneumonia. Journal of Hospital Medicine**.** 2010; 5:528-34.

4. Shorr AF, Tabak YP, Gupta V, Johannes RS, Liu LZ, Kollef MH**.** Morbidity and cost burden of methicillin-resistant Staphylococcus aureus in early onset ventilator-associated pneumonia. Critical care (London, England)**.** 2006; 10:R97.

5. Itani KM, Merchant S, Lin SJ, Akhras K, Alandete JC, Hatoum HT**.** Outcomes and management costs in patients hospitalized for skin and skin-structure infections. American Journal of Infection Control**.** 2011; 39:42-9.

6. Li X, Chen Y, Gao W, Ouyang W, Wei J, Wen Z**.** Epidemiology and outcomes of complicated skin and soft tissue infections among inpatients in southern China from 2008 to 2013. PLoS One**.** 2016; 11:e149960.

7. Park SY, Son JS, Oh IH, Choi JM, Lee MS**.** Clinical impact of methicillin-resistant Staphylococcus aureus bacteremia based on propensity scores. Infection**.** 2011; 39:141-7.

8. Klein EY, Jiang W, Mojica N, Tseng KK, McNeill R, Cosgrove SE*, et al***.** National costs associated with methicillin-susceptible and methicillin-resistant Staphylococcus aureus hospitalizations in the United States, 2010-2014. Clinical Infectious Diseases**.** 2019; 68:22-8.

9. Branch-Elliman W, Lee GM, Golen TH, Gold HS, Baldini LM, Wright SB**.** Health and economic burden of post-partum Staphylococcus aureus breast abscess. PLoS One**.** 2013; 8:e73155.

10. Kopp BJ, Nix DE, Armstrong EP**.** Clinical and economic analysis of methicillin-susceptible and -resistant Staphylococcus aureus infections. The Annals of Pharmacotherapy**.** 2004; 38:1377-82.

11. de Kraker MEA, Davey PG, Grundmann H**.** Mortality and hospital stay associated with resistant Staphylococcus aureus and Escherichia coli bacteremia: estimating the burden of antibiotic resistance in Europe. PLoS Medicine**.** 2011; 8.

12. Ott E, Bange FC, Reichardt C, Graf K, Eckstein M, Schwab F*, et al***.** Costs of nosocomial pneumonia caused by meticillin-resistant Staphylococcus aureus. Journal of Hospital Infection**.** 2010; 76:300-3.

13. Ben-David D, Novikov I, Mermel LA**.** Are there differences in hospital cost between patients with nosocomial methicillin-resistant Staphylococcus aureus bloodstream infection and those with methicillin-susceptible S. aureus bloodstream infection? Infection Control and Hospital Epidemiology**.** 2009; 30:453-60.

14. McHugh CG, Riley LW**.** Risk factors and costs associated with methicillin-resistant Staphylococcus aureus bloodstream infections. Infection Control and Hospital Epidemiology**.** 2004; 25:425-30.

15. Thampi N, Showler A, Burry L, Bai AD, Steinberg M, Ricciuto DR*, et al***.** Multicenter study of health care cost of patients admitted to hospital with Staphylococcus aureus bacteremia: Impact of length of stay and intensity of care. American Journal of Infection Control**.** 2015; 43:739-44.

16. Rubio-Terres C, Garau J, Grau S, Martinez-Martinez L**.** Cost of bacteraemia caused by methicillin-resistant vs. methicillin-susceptible Staphylococcus aureus in Spain: a retrospective cohort study. Clinical Microbiology and Infection**.** 2010; 16:722-8.

17. Reed SD, Friedman JY, Engemann JJ, Griffiths RI, Anstrom KJ, Kaye KS*, et al***.** Costs and outcomes among hemodialysis-dependent patients with methicillin-resistant or methicillin-susceptible Staphylococcus aureus bacteremia. Infection Control and Hospital Epidemiology**.** 2005; 26:175-83.

18. Engemann JJ, Carmeli Y, Cosgrove SE, Fowler VG, Bronstein MZ, Trivette SL*, et al***.** Adverse clinical and economic outcomes attributable to methicillin resistance among patients with Staphylococcus aureus surgical site infection. Clinical Infectious Diseases**.** 2003; 36:592-8.

19. Anderson DJ, Kaye KS, Chen LF, Schmader KE, Choi Y, Sloane R*, et al***.** Clinical and financial outcomes due to methicillin resistant Staphylococcus aureus surgical site infection: a multi-center matched outcomes study. PLoS One**.** 2009; 4:e8305.

20. Song X, Perencevich E, Campos J, Short BL, Singh N**.** Clinical and economic impact of methicillin-resistant Staphylococcus aureus colonization or infection on neonates in intensive care units. Infection Control and Hospital Epidemiology**.** 2010; 31:177-82.

21. Filice GA, Nyman JA, Lexau C, Lees CH, Bockstedt LA, Como-Sabetti K*, et al***.** Excess costs and utilization associated with methicillin resistance for patients with Staphylococcus aureus infection. Infection Control and Hospital Epidemiology**.** 2010; 31:365-73.

22. Nelson RE, Jones M, Liu CF, Samore MH, Evans ME, Graves N*, et al***.** The impact of healthcare-associated methicillin-resistant Staphylococcus aureus infections on post-discharge healthcare costs and utilization. Infection Control and Hospital Epidemiology**.** 2015; 36:534-42.

23. Lee YJ, Chen JZ, Lin HC, Liu HY, Lin SY, Lin HH*, et al***.** Impact of active screening for methicillin-resistant Staphylococcus aureus (MRSA) and decolonization on MRSA infections, mortality and medical cost: a quasi-experimental study in surgical intensive care unit. Critical Care (London, England)**.** 2015; 19:143.

24. Resch A, Wilke M, Fink C**.** The cost of resistance: incremental cost of methicillin-resistant Staphylococcus aureus (MRSA) in German hospitals. European Journal of Health Economics**.** 2009; 10:287-97.

25. Nelson RE, Samore MH, Jones M, Greene T, Stevens VW, Liu CF*, et al***.** Reducing time-dependent bias in estimates of the attributable cost of health care-associated methicillin-resistant Staphylococcus aureus infections: a comparison of three estimation strategies. Medical Care**.** 2015; 53:827-34.

26. Xu B, Yuan H, Yang P**.** Evaluation of economic burden induced by multidrug-resistant bacteria related infections in a tertiary general hospital. Chinese Journal of Experimental and Clinical Infectious Diseases**.** 2017; 11:455-9.

27. Capitano B, Leshem OA, Nightingale CH, Nicolau DP**.** Cost effect of managing methicillin-resistant Staphylococcus aureus in a long-term care facility. Journal of the American Geriatrics Society**.** 2003; 51:10-6.

28. Cosgrove SE, Qi Y, Kaye KS, Harbarth S, Karchmer AW, Carmeli Y**.** The impact of methicillin resistance in Staphylococcus aureus bacteremia on patient outcomes: mortality, length of stay, and hospital charges. Infection Control and Hospital Epidemiology**.** 2005; 26:166-74.

29. Lodise TP, McKinnon PS**.** Clinical and economic impact of methicillin resistance in patients with Staphylococcus aureus bacteremia. Diagnostic Microbiology and Infectious Disease**.** 2005; 52:113-22.

30. Kim CJ, Kim HB, Oh MD, Kim Y, Kim A, Oh SH*, et al***.** The burden of nosocomial Staphylococcus aureus bloodstream infection in South Korea: a prospective hospital-based nationwide study. BMC Infectious Diseases**.** 2014; 14:590.

31. Fu J, Chen B, Wang X, Ye Q, Lu Z, Su C*, et al***.** Case-control study of influence of methicillin-resistant Staphylococcus aureus infection on economic losses and length of hospital stay. Chinese Journal of Nosocomiology**.** 2014; 24:2363-5.

32. Engler-Huesch S, Heister T, Mutters NT, Wolff J, Kaier K**.** In-hospital costs of community-acquired colonization with multidrug-resistant organisms at a German teaching hospital. BMC Health Services Research**.** 2018; 18.

33. Butler AM, Olsen MA, Merz LR, Guth RM, Woeltje KF, Camins BC*, et al***.** Attributable costs of Enterococcal bloodstream infections in a nonsurgical hospital cohort. Infection Control and Hospital Epidemiology**.** 2010; 31:28-35.

34. Ford CD, Lopansri BK, Haydoura S, Snow G, Dascomb KK, Asch J*, et al***.** Frequency, risk factors, and outcomes of vancomycin-resistant Enterococcus colonization and infection in patients with newly diagnosed acute leukemia: different patterns in patients with acute myelogenous and acute lymphoblastic leukemia. Infection Control and Hospital Epidemiology**.** 2015; 36:47-53.

35. Kramer TS, Remschmidt C, Werner S, Behnke M, Schwab F, Werner G*, et al***.** The importance of adjusting for enterococcus species when assessing the burden of vancomycin resistance: a cohort study including over 1000 cases of enterococcal bloodstream infections. Antimicrobial Resistance and Infection Control**.** 2018; 7:133.

36. Cheah AL, Spelman T, Liew D, Peel T, Howden BP, Spelman D*, et al***.** Enterococcal bacteraemia: factors influencing mortality, length of stay and costs of hospitalization. Clinical Microbiology and Infection**.** 2013; 19:E181-9.

37. Lloyd-Smith P, Younger J, Lloyd-Smith E, Green H, Leung V, Romney MG**.** Economic analysis of vancomycin-resistant enterococci at a Canadian hospital: assessing attributable cost and length of stay. Journal of Hospital infection**.** 2013; 85:54-9.

38. Adams DJ, Eberly MD, Goudie A, Nylund CM**.** Rising vancomycin-resistant Enterococcus infections in hospitalized children in the United States. Hospital Pediatrics**.** 2016; 6:404-11.

39. Gearhart M, Martin J, Rudich S, Thomas M, Wetzel D, Solomkin J*, et al***.** Consequences of vancomycin-resistant Enterococcus in liver transplant recipients: a matched control study. Clinical Transplantation**.** 2005; 19:711-6.

40. Webb M, Riley LW, Roberts RB**.** Cost of hospitalization for and risk factors associated with vancomycin-resistant Enterococcus faecium infection and colonization. Clinical Infectious Diseases**.** 2001; 33:445-52.

41. Carmeli Y, Eliopoulos G, Mozaffari E, Samore M**.** Health and economic outcomes of vancomycin-resistant enterococci. Archives of Internal Medicine**.** 2002; 162:2223-8.

42. Nguyen GC, Leung W, Weizman AV**.** Increased risk of vancomycin-resistant Enterococcus (VRE) infection among patients hospitalized for inflammatory bowel disease in the United States. Inflammatory Bowel Diseases**.** 2011; 17:1338-42.

43. Jung E, Byun S, Lee H, Moon SY, Lee H**.** Vancomycin-resistant Enterococcus colonization in the intensive care unit: clinical outcomes and attributable costs of hospitalization. American Journal of Infection Control**.** 2014; 42:1062-6.

44. Puchter L, Chaberny IF, Schwab F, Vonberg RP, Bange FC, Ebadi E**.** Economic burden of nosocomial infections caused by vancomycin-resistant enterococci. Antimicrobial Resistance and Infection Control**.** 2018; 7:1.

45. Jiang HL, Zhou Z, Wang LS, Fang Y, Li YH, Chu CI**.** The risk factors, costs, and survival analysis of invasive VRE infections at a medical center in eastern Taiwan. International Journal of Infectious Diseases**.** 2017; 54:18-24.

46. Pelz RK, Lipsett PA, Swoboda SM, Diener-West M, Powe NR, Brower RG*, et al***.** Vancomycin-sensitive and vancomycin-resistant enterococcal infections in the ICU: attributable costs and outcomes. Intensive Care Medicine**.** 2002; 28:692-7.

47. Song XY, Srinivasan A, Plaut D, Perl TM**.** Effect of nosocomial vancomycin-resistant enterococcal bacteremia on mortality, length of stay, and costs. Infection Control and Hospital Epidemiology**.** 2003; 24:251-6.

48. Maslikowska JA, Walker SAN, Elligsen M, Mittmann N, Palmay L, Daneman N*, et al***.** Impact of infection with extended-spectrum beta-lactamase-producing Escherichia coli or Klebsiella species on outcome and hospitalization costs. Journal of Hospital Infection**.** 2016; 92:33-41.

49. Hu B, Ye H, Xu Y, Ni Y, Hu Y, Yu Y*, et al***.** Clinical and economic outcomes associated with community-acquired intra-abdominal infections caused by extended spectrum beta-lactamase (ESBL) producing bacteria in China. Current Medical Research and Opinion**.** 2010; 26:1443-9.

50. MacVane SH, Tuttle LO, Nicolau DP**.** Impact of extended-spectrum beta-lactamase-producing organisms on clinical and economic outcomes in patients with urinary tract infection. Journal of Hospital Medicine**.** 2014; 9:232-8.

51. Yang Y, Ku C, Lin J, Shang S, Chiu C, Yeh K*, et al***.** Impact of extended-spectrum beta-lactamase-producing Escherichia coli and Klebsiella pneumoniae on the outcome of community-onset bacteremic urinary tract infections. Journal of Microbiology Immunology and Infection**.** 2010; 43:194-9.

52. Apisarnthanarak A, Kiratisin P, Mundy LM**.** Predictors of mortality from community-onset bloodstream infections due to extended-spectrum beta-lactamase-producing Escherichia coli and Klebsiella pneumoniae. Infection Control and Hospital Epidemiology**.** 2008; 29:671-4.

53. Lautenbach E, Patel JB, Bilker WB, Edelstein PH, Fishman NO**.** Extended-spectrum beta-lactamase-producing Escherichia coli and Klebsiella pneumoniae: risk factors for infection and impact of resistance on outcomes. Clinical Infectious Diseases**.** 2001; 32:1162-71.

54. Lee SY, Kotapati S, Kuti JL, Nightingale CH, Nicolau DP**.** Impact of extended-spectrum beta-lactamase-producing Escherichia coli and Klebsiella species on clinical outcomes and hospital costs: a matched cohort study. Infection Control and Hospital Epidemiology**.** 2006; 27:1226-32.

55. Apisarnthanarak A, Kiratisin P, Saifon P, Kitphati R, Dejsirilert S, Mundy LM**.** Risk factors for and outcomes of healthcare-associated infection due to extended-spectrum beta-lactamase-producing Escherichia coli or Klebsiella pneumoniae in Thailand. Infection Control and Hospital Epidemiology**.** 2007; 28:873-6.

56. Tumbarello M, Spanu T, Di Bidino R, Marchetti M, Ruggeri M, Trecarichi EM*, et al***.** Costs of bloodstream infections caused by Escherichia coli and influence of extended-spectrum-beta-lactamase production and inadequate initial antibiotic therapy. Antimicrobial Agents and Chemotherapy**.** 2010; 54:4085-91.

57. Thaden JT, Li Y, Ruffin F, Maskarinec SA, Hill-Rorie JM, Wanda LC*, et al***.** Increased costs associated with bloodstream infections caused by multidrug-resistant gram-negative bacteria are due primarily to patients with hospital-acquired infections. Antimicrobial Agents and Chemotherapy**.** 2017; 61.

58. Apisarnthanarak A, Kiratisin P, Saifon P, Kitphati R, Dejsirilert S, Mundy LM**.** Predictors of mortality among patients with community-onset infection due to extended-spectrum beta-lactamase producing Escherichia coli in Thailand. Infection Control and Hospital Epidemiology**.** 2008; 29:80-2.

59. Apisarnthanarak A, Kiratisin P, Saifon P, Kitphati R, Dejsirilert S, Mundy LM**.** Clinical and molecular epidemiology of community-onset, extended-spectrum beta-lactamase-producing Escherichia coli infections in Thailand: a case-case-control study. American Journal of Infection Control**.** 2007; 35:606-12.

60. Alam MF, Cohen D, Butler C, Dunstan F, Roberts Z, Hillier S*, et al***.** The additional costs of antibiotics and re-consultations for antibiotic-resistant Escherichia coli urinary tract infections managed in general practice. International Journal of Antimicrobial Agents**.** 2009; 33:255-7.

61. Esteve-Palau E, Solande G, Sanchez F, Sorli L, Montero M, Gueerri R*, et al***.** Clinical and economic impact of urinary tract infections caused by ESBL-producing Escherichia coli requiring hospitalization: a matched cohort study. Journal of Infection**.** 2015; 71:667-74.

62. Cornejo-Juarez P, Suarez-Cuenca JA, Volkow-Fernandez P, Silva-Sanchez J, Barrios-Camacho H, Najera-Leon E*, et al***.** Fecal ESBL Escherichia coli carriage as a risk factor for bacteremia in patients with hematological malignancies. Supportive Care in Cancer**.** 2016; 24:253-9.

63. Meng X, Liu S, Duan J, Huang X, Zhou P, Xiong X*, et al***.** Risk factors and medical costs for healthcare-associated carbapenem-resistant Escherichia coli infection among hospitalized patients in a Chinese teaching hospital. BMC Infectious Diseases**.** 2017; 17.

64. Leistner R, Bloch A, Sakellariou C, Gastmeier P, Schwab F**.** Costs and length of stay associated with extended-spectrum β-lactamase production in cases of Escherichia coli bloodstream infection. Journal of global antimicrobial resistance. Journal of Global Antimicrobial Resistance**.** 2014; 3:107-9.

65. Huang W, Qiao F, Zhang Y, Huang J, Deng Y, Li J*, et al***.** In-hospital medical costs of infections caused by carbapenem-resistant Klebsiella pneumoniae. Clinical Infectious Diseases**.** 2018; 672:S225-30.

66. Chen Z, Xu Z, Wu H, Chen L, Gao S, Chen Y**.** The impact of carbapenem-resistant Pseudomonas aeruginosa on clinical and economic outcomes in a Chinese tertiary care hospital: a propensity score-matched analysis. American Journal of Infection Control**.** 2018.

67. Lautenbach E, Weiner MG, Nachamkin I, Bilker WB, Sheridan A, Fishman NO**.** Imipenem resistance among pseudomonas aeruginosa isolates: risk factors for infection and impact of resistance on clinical and economic outcomes. Infection Control and Hospital Epidemiology**.** 2006; 27:893-900.

68. Gasink LB, Fishman NO, Weiner MG, Nachamkin I, Bilker WB, Lautenbach E**.** Fluoroquinolone-resistant Pseudomonas aeruginosa: assessment of risk factors and clinical impact. American Journal of Medicine**.** 2006; 119.

69. Morales E, Cots F, Sala M, Comas M, Belvis F, Riu M*, et al***.** Hospital costs of nosocomial multi-drug resistant Pseudomonas aeruginosa acquisition. BMC Health Services Research**.** 2012; 12.

70. Lautenbach E, Synnestvedt M, Weiner MG, Bilker WB, Vo L, Schein J*, et al***.** Imipenem resistance in Pseudomonas aeruginosa: emergence, epidemiology, and impact on clinical and economic outcomes. Infection Control and Hospital Epidemiology**.** 2010; 31:47-53.

71. Gasink LB, Fishman NO, Nachamkin I, Bilker WB, Lautenbach E**.** Risk factors for and impact of infection or colonization with aztreonam-resistant Pseudomonas aeruginosa. Infection Control and Hospital Epidemiology**.** 2007; 28:1175-80.

72. Eagye KJ, Kuti JL, Nicolau DP**.** Risk factors and outcomes associated with isolation of meropenem high-level-resistant Pseudomonas aeruginosa. Infection Control and Hospital Epidemiology**.** 2009; 30:746-52.

73. Cui N, Cao B, Liu Y, Liang L, Gu L, Song S**.** The impact of imipenem-resistant Acinetobacter baumannii infection on clinical outcomes and medical care costs. Chinese Journal of Infectious Diseases**.** 2012; 30:209-14.

74. Zhen X, Chen Y, Hu X, Dong P, Gu S, Sheng YY*, et al***.** The difference in medical costs between carbapenem-resistant Acinetobacter baumannii and non-resistant groups: a case study from a hospital in Zhejiang province, China. European Journal of Clinical Microbiology & Infectious Diseases**.** 2017; 36:1989-94.

75. Lemos EV, de la Hoz FP, Alvis N, Einarson TR, Quevedo E, Castaneda C*, et al***.** Impact of carbapenem resistance on clinical and economic outcomes among patients with Acinetobacter baumannii infection in Colombia. Clinical Microbiology and Infection**.** 2014; 20:174-80.

76. Lautenbach E, Synnestvedt M, Weiner MG, Bilker WB, Vo L, Schein J*, et al***.** Epidemiology and impact of imipenem resistance in Acinetobacter baumannii. Infection Control and Hospital Epidemiology**.** 2009; 30:1186-92.

77. Lee H, Lee H**.** Clinical and economic evaluation of multidrug-resistant Acinetobacter baumannii colonization in the intensive care unit. Infection & Chemotherapy**.** 2016; 48:174-80.

78. Wu X, Ding L, Wu X**.** Direct economic loss due to healthcare-associated infection with multidrug resistant Acinetobacter baumannii. Chinese Journal of Infection Control**.** 2018; 17:735-8.

79. Guo Y, Guo W, Qiu P, He Q, Pan C, Wu C*, et al***.** Study of attributive hospitalized cost and length of stay for hospital acquired infection due to multidrug resistance and none-resistance Acinetobacter baumannii. Chinese Journal of Health Statistics**.** 2017; 34:378-81.

80. Lee NY, Lee HC, Ko NY, Chang CM, Shih HI, Wu CJ*, et al***.** Clinical and economic impact of multidrug resistance in nosocomial Acinetobacter baumannii bacteremia. Infection Control and Hospital Epidemiology**.** 2007; 28:713-9.

81. Thatrimontrichai A, Techato C, Dissaneevate S, Janjindamai W, Maneenil G, Kritsaneepaiboon S*, et al***.** Risk factors and outcomes of carbapenem-resistant Acinetobacter baumannii ventilator-associated pneumonia in the neonate: a case-case-control study. Journal of Infection and Chemotherapy**.** 2016; 22:444-9.

82. Young LS, Sabel AL, Price CS**.** Epidemiologic, clinical, and economic evaluation of an outbreak of clonal multidrug-resistant Acinetobacter baumannii infection in a surgical intensive care unit. Infection Control and Hospital Epidemiology**.** 2007; 28:1247-54.

83. Wilson SJ, Knipe CJ, Zieger MJ, Gabehart KM, Goodman JE, Volk HM*, et al***.** Direct costs of multidrug-resistant Acinetobacter baumannii in the burn unit of a public teaching hospital. American Journal of Infection Control**.** 2004; 32:342-4.
